# Two-phase wash to solve the ubiquitous contaminant-carryover problem in commercial nucleic-acid extraction kits

**DOI:** 10.1038/s41598-020-58586-3

**Published:** 2020-02-06

**Authors:** Erik Jue, Daan Witters, Rustem F. Ismagilov

**Affiliations:** 1 Division of Biology and Biological Engineering, California Institute of Technology 1200 E. California Blvd., Pasadena, CA 91125 United States; 2 Division of Chemistry and Chemical Engineering California Institute of Technology 1200 E. California Blvd., Pasadena, CA 91125 United States

**Keywords:** Molecular biology, Biological techniques, Isolation, separation and purification

## Abstract

The success of fundamental and applied nucleic acid (NA) research depends on NA purity, but obtaining pure NAs from raw, unprocessed samples is challenging. Purification using solid-phase NA extractions utilizes sequential additions of lysis and wash buffers followed by elution. The resulting eluent contains NAs and carryover of extraction buffers. Typically, these inhibitory buffers are heavily diluted by the reaction mix (e.g., 10x dilution is 1 µL eluent in 9 µL reaction mix), but in applications requiring high sensitivity (e.g., single-cell sequencing, pathogen diagnostics) it is desirable to use low dilutions (e.g., 2x) to maximize NA concentration. Here, we demonstrate pervasive carryover of inhibitory buffers into eluent when several commercial sample-preparation kits are used following manufacturer protocols. At low eluent dilution (2–2.5x) we observed significant reaction inhibition of polymerase chain reaction (PCR), loop-mediated isothermal amplification (LAMP), and reverse transcription (RT). We developed a two-phase wash (TPW) method by adding a wash buffer with low water solubility prior to the elution step. The TPW reduces carryover of extraction buffers, phase-separates from the eluent, and does not reduce NA yield (measured by digital PCR). We validated the TPW for silica columns and magnetic beads by demonstrating significant improvements in performance and reproducibility of qPCR, LAMP, and RT reactions.

## Introduction

Polymerase chain reaction (PCR) is a widely used tool in molecular biology for generating many nucleic acid (NA) copies from a starting DNA template. PCR may also be combined with reverse transcription (RT) to amplify many DNA copies from a starting RNA template. The amplified NAs then serve different purposes, such as detection, quantification, library preparation for sequencing, or generating constructs for cloning^1,2^. NA amplification is crucial in highly sensitive applications (few DNA copies) such as single-cells analyses or the detection of SNPs, cell-free circulating DNA, or pathogens^3–5^. Isothermal amplifications are an attractive alternative to PCR that eliminate the stringent temperature cycling requirements^6^. Specifically, loop-mediated isothermal amplification (LAMP) is faster than PCR and is especially promising for diagnostic devices in point-of-care settings^7,8^. PCR, RT, and LAMP typically require purified NAs as starting template; however, extracting purified NAs from raw, unprocessed samples is challenging^9^. Though commonly overlooked, the efficient and effective extraction of pure NAs is of paramount importance^10^.

A primary function of NA extractions is to eliminate inhibitors. If inhibitors are transferred into the eluent, they can delay or completely inactivate downstream applications such as PCR and LAMP^11,12^. Inhibitors have also been implicated in failed RT, molecular cloning, and sequencing experiments^13–15^. We anticipate two potential sources of inhibitors: (1) those present in the raw, unprocessed sample and (2) those introduced during the NA extraction^16^. There have been numerous studies demonstrating the adverse effects of inhibitors in challenging sample matrices, such as humic acids, food particles, cellular debris, urine, blood, and stool^11,12,17–25^. To remove these inhibitors, solid-phase extractions are an effective choice because they have been found to yield higher purity compared with other extraction methods^19,20,26–29^. The two most common solid-phase extraction methods use either spin columns or magnetic beads^28,30^. In both methods, the sample is first mixed with a lysis/binding buffer, the lysed sample contacts the solid phase allowing NAs to bind, the solid phase is cleansed with one or more wash buffers, and the NAs are eluted with water. Typically, the lysis/binding buffer contains a chaotropic salt (e.g., guanidinium isothiocyanate) whereas the wash buffer contains a high concentration of ethanol (or isopropanol). Any carryover of these extraction buffers (lysis buffer or wash buffer) into the eluent could be greatly inhibitory to downstream analyses.

The purified eluent contains NAs and any carried-over extraction buffers at their highest concentration. To run a downstream reaction, a volume of eluent is mixed with a volume of reaction mix. For research applications, it is standard to dilute the eluent 10x (e.g., 1 µL eluent and 9 µL reaction mix)^31,32^, 25x (e.g., 1 µL eluent and 24 µL reaction mix)^33^, or more^34,35^. At these high eluent dilutions, concentrations of inhibitors present in the eluent are reduced and thus their potential negative effects on the reaction are mitigated. However, the dilution of inhibitors equally dilutes the NAs, which may be detrimental when the original sample has low NA concentrations^3^ and/or when high sensitivity is desired. For example, single nucleotide polymorphisms^5^, cell-free circulating DNA^4^, and single-cell analyses all require maximizing the concentration of NA loaded into the amplification mix. Maximizing NA concentration is also important for infectious disease diagnostics and monitoring the water supply, food supply, and environment^32,36–38^. For these applications, a higher NA concentration could be achieved with a lower dilution (e.g., a 2.5x dilution would be 4 µL eluent and 6 µL reaction mix). The theoretical maximum NA concentration could be attained by eliminating the dilution altogether, which is only possible by adding eluent directly to a dried reaction mix (e.g., 10 µL eluent and dry reaction mix to make ~10 µL reaction). This can be achieved with lyophilization, wherein reagents are freeze-dried to a powder, or other approaches for generating dry reaction mixes. The use of dry reagents has additional benefits: simple assay protocols, lenient reagent-storage conditions, and long reagent shelf-life, all of which are desirable characteristics for the development of point-of-care devices. However, in using low dilutions or no dilution, extraction buffers in the eluent are used at higher concentrations which may have adverse effects on downstream reactions.

Few studies have directly investigated inhibition resulting from solid-phase extraction kit buffers^39,40^. In this manuscript, we aimed to quantify and reduce inhibition arising from buffer carryover in commercial extraction kits from well-known suppliers. We first identified that kit buffer carryover is indeed a concern when using low eluent dilutions (≤2.5x) for both commercial silica-column and magnetic-bead extractions (following manufacturer protocols). To improve our understanding of inhibition, we performed a detailed study using a range of buffer dilutions from different extraction kits. To address the carryover of kit buffers, we developed modified extraction protocols utilizing an additional two-phase wash (TPW) that would integrate easily with the existing manufacturer protocols^41^. The TPW is a compound with low water solubility, can be added in between the wash and elution steps, and it phase-separates with water after the elution step. We identified an optimized set of TPW candidates among several potential compounds and then evaluated TPW performance by testing kit protocols from leading manufacturers (Zymo and Qiagen) at both low and high eluent dilutions. To unambiguously show that inhibition is due to kit buffer inhibitors, as opposed to sample inhibitors or losses of NAs, we performed extractions on pure water samples with or without the TPW, and added the resulting kit extract to spiked qPCR, LAMP, and RT assays.

## Materials and Methods

### NA stocks and primers

Lambda (λ) phage DNA (linear double-stranded 500 µg/mL, N3011L, New England Biolabs (NEB)) was purchased from NEB and the stock was quantified at 1.1 × 10^10^ cp/µL using digital PCR (dPCR). *Escherichia coli* DNA was extracted from an NEB 5-alpha strain using Epicentre QuickExtract DNA Extraction Buffer (Lucigen Corporation,Middleton, WI, USA) and the stock was quantified at 1.4 × 10^7^ cp/µL using dPCR. *Neisseria gonorrhoeae* live infectious stock (Z017, Zeptometrix, Buffalo, NY, USA) was resuspended to 5 × 10^7^ cfu/mL in pre-warmed (37 °C) Hardy Diagnostics FB Broth (K31, Hardy Diagnostics, Santa Maria, CA, USA) and diluted an additional 10-fold in urine to 5 × 10^6^ cfu/mL. Urine from healthy human donors (>18 years of age) was acquired and used in accordance with approved Caltech Institutional Review Board (IRB) protocol 15–0566. Informed consent was obtained from all participants. Urine sample donations were never tied to personal identifiers and all research was performed in accordance with the approved IRB protocol and relevant institutional biosafety regulations. Urine samples were stored at room temperature and used within 1 h of collection. Spiked urine (125 µL) was mixed with DNA/RNA Shield (125 µL) and lysis buffer (500 µL) for a total lysed sample volume of 750 µL. Both DNA and RNA were extracted simultaneously with a ZR Viral DNA/RNA Kit, and *N. gonorrhoeae* 16S RNA was found to be in over 200-fold excess of 16S DNA as verified by dPCR with or without an RT step. All NA stocks were diluted at least 100-fold into all reactions, thereby eliminating the effects of any inhibitors that could be present in the NA stock. Lambda LAMP primers^42^, Lambda PCR primers^43^, *E. coli* 23S rRNA gene LAMP primers^44^, *E. coli* 23S rRNA gene PCR primers^45^, and *N. gonorrhoeae* 16S rRNA gene PCR primers^46^ have been previously published and were supplied by Integrated DNA Technologies using standard desalting purification.

### Kit extractions

We tested three different silica-column kits: Zymo ZR Viral DNA/RNA Kit (outdated protocol, D7021), Zymo Quick-DNA/RNA Kit (updated protocol, D7021), and the QIAquick PCR Purification Kit (28104, Qiagen). For all silica-column kits, fresh collection tubes were used after each spin and centrifugation speeds were set to 16,000 × g. Centrifugation was performed on either an Eppendorf 5415D centrifuge (Eppendorf, Hauppauge, NY, USA) or a Thermo Fisher Scientific AccuSpin Micro 17 R centrifuge (13–100–676). We note that the QIAquick protocol calls for 17,900 × g, but we instead ran at 16,000 × g which was the max speed for the Eppendorf 5415D. For both Zymo kits, 750 µL lysed sample was prepared by mixing 125 µL sample with 125 µL Zymo 2x DNA/RNA Shield and 500 µL Viral DNA/RNA Buffer. For the Zymo ZR Viral DNA/RNA kit, 750 µL lysed sample was centrifuge for 1 min, 500 µL Zymo Viral Wash Buffer was centrifuged for 2 min, and 50 µL nuclease-free water was centrifuged for 30 s into a clean 1.5 mL tube. Optionally, either a dry spin or 300 µL TPW was centrifuged for 2 min in between the Viral Wash Buffer and elution steps. For the Zymo Quick-Viral DNA/RNA kit, 750 µL lysed sample was centrifuged for 1 min, 500 µL Zymo Viral Wash Buffer was centrifuged for 30 s, an additional 500 µL Zymo Viral Wash Buffer was centrifuged for 30 s, 500 µL 200 proof ethanol was centrifuged for 1 min, and 50 µL nuclease-free water was centrifuged for 30 s into a clean 1.5 mL tube. Optionally, either a dry spin or 300 µL TPW was centrifuged for 1 min in between the ethanol and elution steps. For the QIAquick PCR Purification Kit, 125 µL sample was mixed with 625 µL Buffer PB without indicator. 750 µL lysed sample was centrifuged for 30 s, followed by 750 µL Buffer PE for 30 s, a dry spin for 1 min, and 50 µL nuclease-free water for 1 min. Optionally, the dry spin was skipped or the dry spin was replaced with a 300 µL TPW and centrifuged for 1 min.

We tested the Zymo Quick-DNA/RNA Viral MagBead kit (R2140). For the Zymo MagBead kit, 200 µL sample was mixed with 200 µL Zymo 2x DNA/RNA Shield, 4 µL Proteinase K, and 800 µL Zymo Viral DNA/RNA Buffer. 1204 µL was added to each tube, mixed with 20 µL MagBinding Beads, and placed on an UltraRocker Rocking Platform (1660709EDU, Bio-Rad, Hercules, CA, USA) for 10 min at max speed. Tubes were transferred to a DynaMag-2 magnetic rack (12321D, Thermo Fisher Scientific) and we followed manufacturer instructions for the remainder of the protocol. Optionally, the 10 min dry step was skipped or the dry step was instead replaced with the addition of 500 µL TPW. In the modified protocol for the Zymo MagBead kit, we waited at least one additional minute and perform a second aspiration after each aspiration step in the manufacturer’s protocol.

### qPCR mix

qPCR reactions contained 1X Bio-Rad SsoFast Supermix (1725201, Bio-Rad), PCR primers (IDT) at 0.5 µM each, and were supplemented with nuclease-free water up to 10 µL. Each 96-well plate (thin-wall clear well, HSP9641, Bio-Rad) was sealed (Microseal B, MSB1001, Bio-Rad) and briefly spun in a Mini Plate Spinner Centrifuge (14-100-141, Fisher Scientific). Heating and real-time imaging were performed on the Bio-Rad CFX-96 Touch Real-Time PCR Detection System by heating to 95 °C for 5 min, cycling 40 times between 95 °C for 15 s, 60 °C for 15 s, and 72 °C for 20 s, and taking a melt-curve analysis. For the *E. coli* DNA dilution experiment, qPCR was run for 60 cycles. Fluorescence readings were taken at the end of each extension step. Quantification cycle (C_q_) was determined when the software’s automated baseline corrected fluorescence reached 200 RFU.

### LAMP mix

LAMP reactions contained the following concentrations of reagents: 1X Isothermal Amplification Buffer (20 mM Tris-HCl pH 8.8, 10 mM (NH_4_)_2_SO_4_, 50 mM KCl, 5 mM MgSO_4_, 0.1% Tween-20, B0537S, NEB, Ipswich, MA, USA), an additional 2 mM MgSO_4_ (B1003S, NEB), 1.4 mM deoxynucleotide mix (N0447L or N0446S, NEB), 2 µM Invitrogen Syto-9 (S34854, Thermo Fisher Scientific), 2 µM Invitrogen bovine serum albumin (15561020, Thermo Fisher Scientific), 320 U/mL WarmStart Bst 2.0 (M0538L, NEB), and were supplemented with nuclease-free water (not DEPC-Treated, 4387936, Thermo Fisher Scientific) up to 10 µL. LAMP primers (Integrated DNA Technologies (IDT), Coralville, IA, USA) were designed, ordered, and added at NEB’s recommended concentrations of 1.6 µM FIP/BIP, 0.2 µM F3/B3, and 0.4 µM LoopF/B. Each 96-well plate was sealed and briefly spun. Heating and real-time imaging were performed on the Bio-Rad CFX-96 Touch Real-Time PCR Detection System (1855195, Bio-Rad). Each 96-well plate was cooled to 12 °C for 2 min, held at 68 °C for 47 min with 35-second fluorescence read intervals, and we performed a melt-curve analysis. For the *E. coli* DNA dilution experiment, the 68 °C step was held for 105 min. Time-to-positive (TTP) was determined when the software’s automated baseline corrected fluorescence reached 1000 RFU.

### Buffer inhibition

For studying kit buffer inhibitors, LAMP and qPCR reactions were spiked to 5 × 10^4^ cp/rxn λ phage DNA (NEB) and supplemented with half-log dilutions of either Koptec 200-proof ethanol (V1001, Decon Labs, King of Prussia, PA, USA), Viral RNA Wash Buffer 1x (R1034-2-48, Zymo Research, Tustin, CA, USA), Buffer PE (19065, Qiagen, Germantown, MD, USA), Zymo DNA/RNA Shield 1x (R1200-125), Zymo Viral DNA/RNA Buffer (D7020-1-100), or Qiagen Buffer PB (19066) to the appropriate final concentration. For selecting the optimal TPW, LAMP and qPCR reactions were spiked with 1 µL of 5 × 10^4^ cp/µL λ phage DNA, diluted to 10 µL, and an additional 1 µL was added of either nuclease-free water, 200 proof ethanol, isopropanol (BP2618-500, Thermo Fisher Scientific, Waltham, MA, USA), 1-butanol (3000-04, Mallinckrodt Chemicals), isopentanol (2992-04, Mallinckrodt Chemicals), 1-hexanol (H13303–100 mL, MilliporeSigma, St. Louis, MO, USA), 1-heptanol (H2805-250 mL, MilliporeSigma), 1-octanol (SHBH2844V, MilliporeSigma), 1-nonanol (131210–100 mL, MilliporeSigma), 1-decanol (2397563–50 g, MilliporeSigma), 1-undecanol (MKCG3271, MilliporeSigma), 2-dodecanol (D221503-5G, MilliporeSigma), 5 cSt silicone oil (317667-250 mL, MilliporeSigma), or Fluorinert FC-40 (ZF-0002-1308-0, 3 M, St. Paul, MN, USA).

### dPCR mix

Droplet digital PCR (dPCR) experiments were performed on a Bio-Rad QX200 Droplet Digital PCR System (1864001, Bio-Rad). dPCR mixes were made with 1X QX200 dPCR EvaGreen Supermix (1864034, Bio-Rad), 200 nM forward primer, and 200 nM reverse primer. Eluent was diluted 10x in separate tubes and an additional 10x into the reaction mix. All samples were made to 50 µL and duplicates were run by adding 22 µL to two sample wells in the DG8 Cartridge for droplet generator (1864008, Bio-Rad). Droplet generation, droplet transfer, and foil sealing followed manufacturer’s instructions. Thermocycling took place on a C1000 Touch Thermal Cycler (Bio-Rad) with a pre-melt at 95 °C for 3 min, 40 cycles of 95 °C for 30 s, 60 °C for 30 s, and 68 °C for 30 s, and a stabilization at 4 °C for 5 min, 90 °C for 5 min, and a hold at 12 °C until droplet analysis. A temperature ramp rate of 2 C/s was used for temperature transitions. Droplets were read according to manufacturer instructions. Analysis thresholds were manually set at the valley between negative and positive droplets. Final concentrations were determined using the merge setting on the QuantaSoft analysis software. No template controls (NTC) were always run and showed negligible normalized counts (<0.1%).

### RT mix

The RT reaction contained 1X Isothermal Amplification Buffer, 0.5 mM dNTP Mix, 0.2 µM primers, 1 U/µL Riboguard RNase Inhibitor (RG90910K, Lucigen, Middleton, WI, USA), and 0.15 U/µL WarmStart Rtx (M0380L, NEB). The extracted *N. Gonorrhoeae* RNA was diluted 10x in a separate tube and an additional 10x by adding 2.5 µL into the 25 µL reaction mix (100x dilution total). Kit extracts were spiked in the reaction mix by adding either 2.5 µL (10x) or 12.5 µL (2x). We added water to a total reaction volume of 25 µL. Temperature was set to anneal for 5 min at 25 °C, incubate for 10 min at 55 °C, and inactivate for 10 min at 80 °C in a C1000 Touch Thermal Cycler (1851196, Bio-Rad).

## Results and Discussion

### Establishing the presence and prevalence of inhibitors in buffers

We first carefully designed an experiment to evaluate the presence, prevalence, and effects of buffer carryover when using standard commercial NA extraction kits. To eliminate the confounding effects of NAs or inhibitors originating from the sample, we performed NA extractions on pure water samples (Fig. S1). When extracting from pure water samples, we refer to the eluent as the “kit extract,” which only contains water and inhibitors originating from buffers in the extraction kits. Here, we tested a centrifugation-based NA extraction using a Zymo ZR Viral DNA/RNA Kit and followed the manufacturer’s protocol. Next, we mixed the kit extract into a qPCR reaction spiked with *λ* phage DNA at either a 10x dilution (1 µL kit extract, 0.5 µL template DNA, 8.5 µL reaction mix) or 2.5x dilution (4 µL kit extract, 0.5 µL template, 5.5 µL reaction mix). We used heavily diluted purified *λ* phage DNA to ensure no inhibition originated from the template. The 10x and 2.5x dilution reactions contain different volumes of kit extract, but each had a final volume of 10 µL and contained the same concentration of *λ* phage template, *λ* phage primers, and qPCR components. We ran qPCR on a thermocycler for 40 cycles while taking readings at the end of each cycle. If the kit extracts have no inhibitory effect, we would expect the same quantification cycle (C_q_) for both reactions. Given the amount of input DNA (5 × 10^4^ copies), we expect amplification to occur at ~20 cycles.

**Figure 1:**
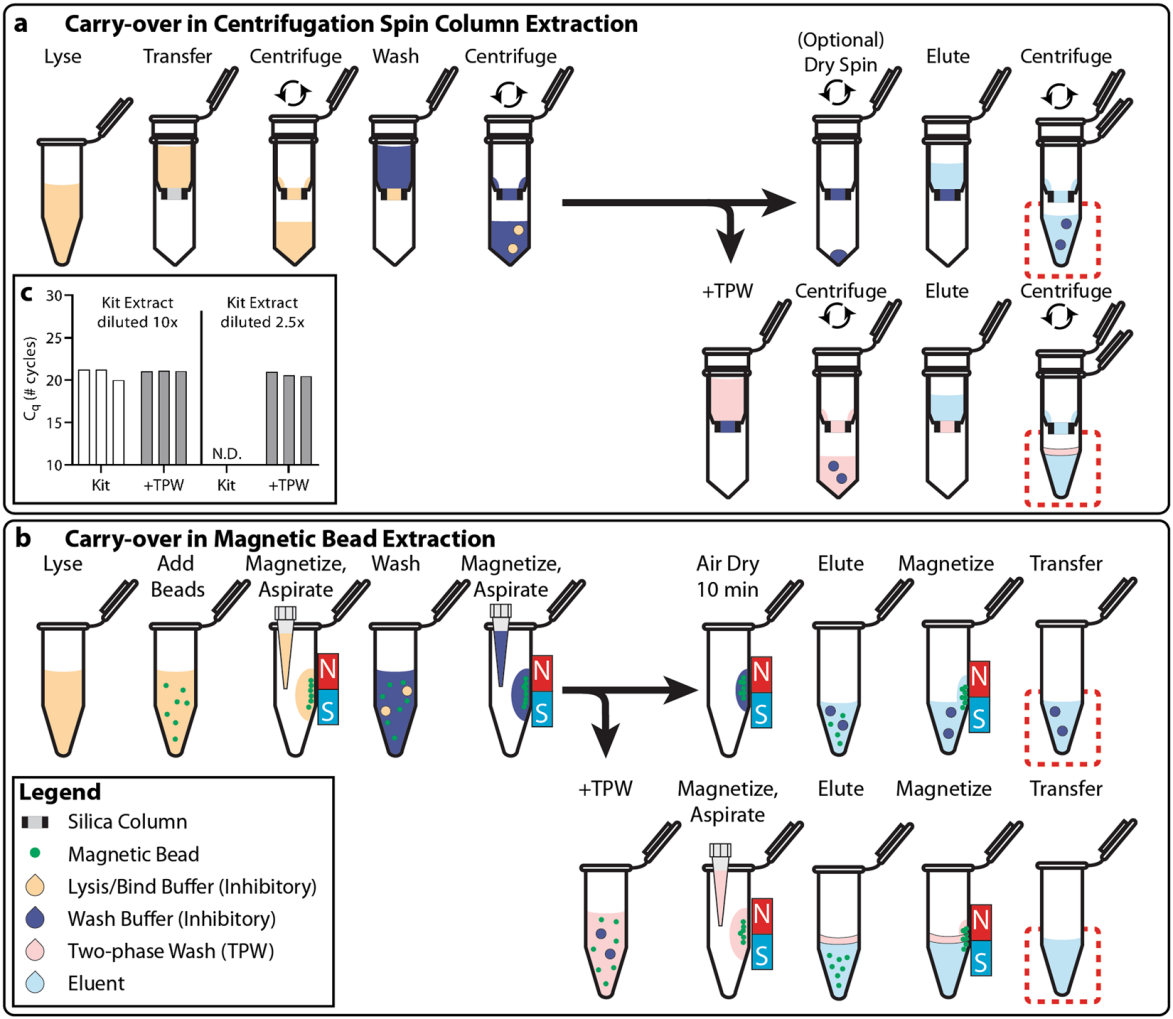
Schematic depicting the carryover of buffers during sample preparation when nucleic acids (NA) are extracted using either (**a**) spin column centrifugation or (**b**) magnetic beads. Dashed red boxes highlight carryover of buffer into the eluent. Carryover buffer from the previous wash either mixes with the eluent (top dashed box in each panel) or phase separates (bottom dashed box in each panel) when the two-phase wash (TPW) is used. (**c**) Inset graph shows a qPCR run spiked with 5 × 10^4^ copies λ phage DNA and λ phage primers into which we added Zymo ZR “kit extract.” (When extracting from pure water samples, we refer to the eluent as the “kit extract,” which only contains water and inhibitors originating from buffers in the extraction kits). The graph compares the reaction inhibition in a 10x extract dilution and a 2.5x extract dilution and shows the effect of adding a TPW (+TPW) during the nucleic-acid extraction step. Inhibition is similarly observed for magnetic bead extraction kits. N.D. stands for not detected. We ran 6 extractions (3 silica columns × 2 conditions) and used the same kit extract to make the high- and low-dilution conditions.

Using the centrifugation sample-preparation protocol (Fig. 1a) and a 2.5x dilution of kit extract, amplification in qPCR was completely inhibited (Fig. 1c). In contrast, using the 10x dilution, all three kit extracts (three separate columns) amplified at ~20 cycles as expected. The only variable that differed between the two conditions was that the 2.5x dilution (4 µL kit extract) contained four times the concentration of buffer compared with the 10x dilution (1 µL kit extract). This result led us to conclude that carryover of inhibitory buffers is inhibiting the qPCR reaction.

We suspect that carryover results from residual buffer trapped in the column that is picked up during elution. Although centrifugation moves most of the extraction buffers to the waste tube for removal, some lysis/binding buffer and/or wash buffers may remain stuck in the column after each centrifugation step (Fig. 1a). This could occur due to capillary pressure, physical entrapment, surface tension, or physicochemical interactions with either the silica column or the walls of the tube. Furthermore, it is possible for some of the inhibitory components contained in the buffer to become unevenly trapped on the column. During the elution step, water could mix with these trapped buffers/inhibitors and carry them into the final eluent. We emphasize that for a standard elution volume of 50 µL water, even low volumes of carryover may correspond to a sufficiently inhibitory percentage of buffer in the eluent. For example, 500 nL buffer carryover corresponds to 1% buffer in the eluent and 2.5 µL corresponds to 5% buffer in the eluent.

Buffer carryover also occurred when using magnetic-bead extraction. In these protocols, magnetic beads that bind to NAs in the appropriate buffer conditions are added to the sample. Extraction buffers are then added (lysis and multiple washes) by sequential rounds of buffer addition, magnetization to pull the magnetic beads to the side of the tube, and aspiration of each buffer (Fig. 1b). For the elution step, water is added which releases the NAs from the magnetic beads, the magnetic beads are drawn to the sides of the tube, and the eluent is transferred to a clean tube. During this process, however, some buffer components may stick to the magnetic beads or adhere to the walls of the tube. Thus, although most of the buffers are removed during aspiration, a low concentration of extraction buffers transfer into the eluent when using the standard manufacturer protocols. Below (section “TPW validation for magnetic-bead extractions”), we explicitly examine the extent of buffer carryover for magnetic-bead extractions using low and high dilutions of eluent.

We hypothesized that we could address the issue of extraction buffer carryover in commercial NA extraction kits by the addition of a TPW. The TPW is composed of an immiscible compound that phase separates with water, and we added it in between the wash step and the final elution (Fig. 1a bottom, 1b bottom). Our aim was to develop a TPW that would be simple, inexpensive, and that would integrate easily with existing protocols. If successful, the TPW would greatly reduce buffer carryover and improve downstream assay performance. In our study (Fig. 1c), incorporating the TPW recovered qPCR (2.5x dilution of kit extract) and provided the expected Cq of ~20 cycles. This was a drastic performance improvement compared with the complete reaction inhibition we observed when the same dilution was run using the manufacturer protocol.

### Exploring the effects of buffer inhibition on amplification

Having established that buffer carryover is a problem, we next aimed to better understand the effects of inhibition on amplification in qPCR and LAMP. We selected extraction buffers from a Zymo viral DNA/RNA kit and a Qiagen PCR purification kit. We chose these two commercial kits in particular because they both utilize minimal protocols (lysis, wash, elute) with no added steps (e.g. bacterial pellet spins, proteinase K, lysozyme, DNase/RNase, filtration, etc.). Specifically, we wanted to identify the concentration at which each buffer inhibits qPCR and LAMP. First, we added buffers at half-log dilutions (from 10% down to 0.032%) into *λ* phage spiked qPCR or LAMP reactions (1 µL diluted buffer, 1 µL template, 8 µL reaction mix). We were also curious to see whether qPCR and LAMP were affected differently by inhibitors. We expected differences between the two amplification methods because qPCR amplification is temperature-gated whereas LAMP amplifies continuously. Previous literature on this topic shows “mixed results;” many studies have shown that LAMP is more robust than PCR in the presence of inhibitors^47–50^ whereas others have shown that inhibition of PCR and LAMP depends on which inhibitor was used^40^.

**Figure 2:**
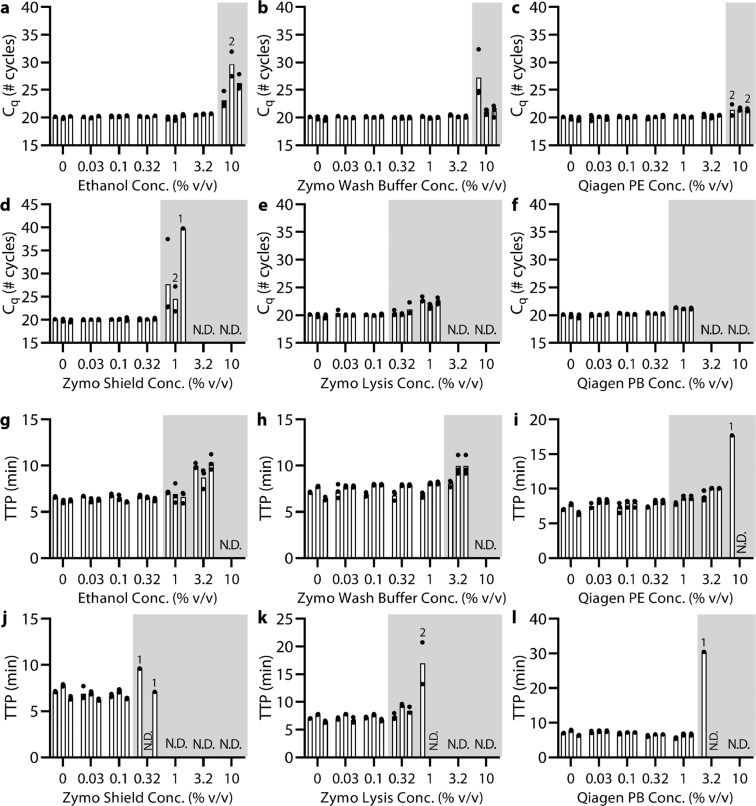
(**a**–**f**) qPCR and (**g**–**l**) LAMP experiments demonstrate reaction inhibition from NA extraction kit buffers. Quantification cycles (C_q_) for qPCR or time to positive (TTP) for LAMP spiked with 5 × 10^4^ copies λ phage DNA and primers with increasing concentrations of extraction kit buffers. For ethanol dilutions (**a**,**g**), three separate amplification mixes were each combined with an independent ethanol dilution series. All remaining buffer dilutions (**b**–**f**,**h**–**l**) shared the same set of three amplification mixes (same 0% condition), and each amplification mix was combined with an independent dilution series of each buffer. Each bar is the average of qPCR or LAMP technical triplicates (black circles). Where shown, numbers above a bar indicate the number of samples that amplified out of technical triplicates. Gray shading indicates when inhibition (>0.5 cycles or >0.5 min) was observed according to changes in C_q_ or TTP. Samples marked N.D. were not detected within either 40 cycles or 40 min.

We found that all extraction buffers were inhibitory to both types of reactions, but at different concentrations (Fig. 2). As a control, for each kit, we ran the protocol with 0% buffer and found amplification with qPCR to yield a C_q_ of ~20.0 ± 0.3 cycles and amplification with LAMP to have a TTP of 7.1 ± 0.6 min. As a general trend, we found that wash buffers (ethanol, Zymo Viral Wash Buffer, and Qiagen Buffer PE; Fig. 2a–c,g–i) were less inhibitory than lysis buffers (Zymo DNA/RNA Shield, Zymo DNA/RNA Viral Buffer, and Qiagen Buffer PB; Fig. 2d–f,j–l). For qPCR, we observed a statistically significant (*P* < 0.05) C_q_ delay of at least 0.5 cycles for wash buffer concentrations starting at 10% (Fig. 2a–c, Table S1) and for lysis buffers starting between 0.32–1% (Fig. 2d–f, Table S1). For LAMP, we observed a statistically significant (*P* < 0.05) TTP delay of at least 0.5 min for wash buffer concentrations starting at 1–3.2% (Fig. 2g–i, Table S2) and for lysis buffers starting at 0.32–3.2% (Fig. 2j–l, Table S2). These results imply that the extent of inhibition on qPCR and LAMP reactions is inhibitor-dependent, which may help explain the “mixed results” in the literature.

Next, we observed the presence of inhibitors at very low concentrations using melting temperature (Tm), as compared with C_q_, TTP, or endpoint fluorescence (Figs. S1–S4). Interestingly, we observed that the presence of extraction buffers raised or lowered the Tm of the DNA product even at very low concentrations (1–3.2% for ethanol buffers, 0.32–1% for lysis buffers). Detecting a change in the Tm of an NA product could be a useful tool for diagnosing the presence or absence of extraction buffers in a reaction.

### Inhibition in samples with low NA concentrations

We next wished to test the effects of buffer-related inhibition in samples containing low NA concentrations. For applications requiring high sensitivity (e.g., single-cell sequencing, cell-free circulating DNA, SNP genotyping, and diagnostics), amplification reactions are often run at or near the limit-of-detection (LOD). Samples starting with low NA concentrations thus require the polymerase to replicate more DNA than in samples that start with a high NA concentration. Therefore, we hypothesized that the inhibition effect resulting from buffer carryover would be stronger for these low NA samples (and detected as delayed C_q_ or TTP). Additionally, it has been recorded that PCR reactions with different primers and targets can respond differentially to inhibitors^11^. To ensure the inhibitory effects we saw with *λ* phage DNA were not specific to just the set of DNA and primers we used, we ran this experiment using *Escherichia coli* DNA and *E. coli* primers.

**Figure 3:**
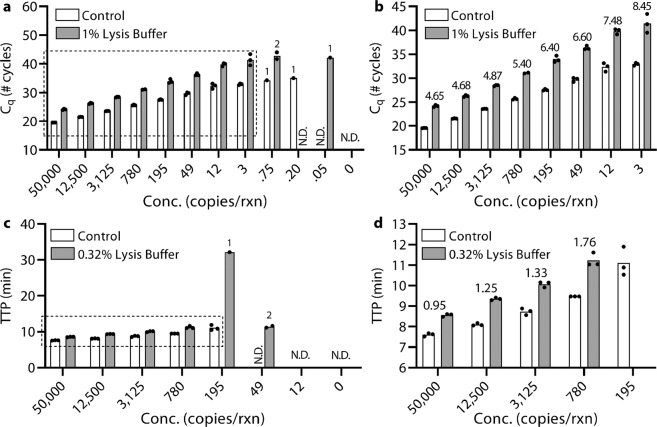
(**a**,**b**) qPCR and (**c**,**d**) LAMP experiments targeting *E. coli* 23S rRNA gene, which shows increased impact of reaction inhibition at low NA concentrations. (**a**) qPCR and (**c**) LAMP spiked with 4-fold dilution series of *E. coli* 23S rRNA gene copies and comparing with and without Zymo Viral DNA/RNA Buffer. Each bar represents the average of technical qPCR or LAMP triplicates (black circles). Numbers above a bar indicate the number of samples which amplified if not all triplicates were detected. Dashed boxes indicate axes for zoomed-in graphs of (**b**) qPCR and (**d**) LAMP. Numbers above each pair of bars indicate the difference in either C_q_ or TTP between the control and the reaction with added lysis buffer. Samples marked N.D. were not detected within either 60 cycles or 40 min.

With qPCR, we found that the cycle delay as a result of buffer inhibitors was higher at lower NA concentrations (Fig. 3a,b). We started with a medium concentration of target (5 × 10^4^ *E. coli* 23S copies) and tested 4-fold dilutions down to 0.05 copies with either control (no inhibition) or in the presence of 1% Zymo Viral DNA/RNA Buffer. We chose 1% lysis buffer because we had found 1% lysis buffer to be weakly inhibitory and we suspected inhibition may worsen with decreasing DNA concentration.

Our control reactions matched our expectations; we found 5 × 10^4^ copies yielded a C_q_ of 19.55 ± 0.04, the cycle increased by ~2 for every 4-fold dilution, and we detected the target down to 3 copies. Compared with the 1% lysis buffer condition, we found that the reaction for the highest concentration (5 × 10^4^ copies) was greatly impaired by 4.65 ± 0.13 (95% CI: 4.33–4.97) cycles (Fig. 3b). The delay worsened and variance increased as the NA concentration was decreased. At 3 copies/rxn, there was an 8.45 ± 0.94 (95% CI: 6.11–10.79) cycle delay and all three triplicates amplified, but we needed to increase the number of cycles in this experiment in order to detect the delayed C_q_. Our results showed that the presence of lysis buffer caused a decrease in the amplification efficiency with each cycle. This conclusion was also supported by the shallower amplification curves (Fig. S7).

With LAMP, we also found that the delay as a result of buffer inhibitors was higher at lower NA concentrations (Fig. 3c,d). Because LAMP was more sensitive to inhibitors than qPCR, we compared the control to 0.32% lysis buffer. The control reaction TTP was 7.61 ± 0.08 min at 5 × 10^4^ copies and the TTP increased with increasing dilutions up to 11.1 ± 0.7 min at 195 copies. LAMP failed to amplify at higher concentrations of DNA than when using qPCR (amplification for 3 or fewer copies was stochastic). The addition of 0.32% lysis buffer caused a 0.95 ± 0.06 (95% CI: 0.80–1.10) min delay in TTP at the highest concentration (5 × 10^4^ copies/rxn), which increased as the *E. coli* DNA concentration was lowered to a 1.76 ± 0.19 (95% CI: 1.29–2.23) min delay at the lowest detectable concentration (780 copies/rxn). At lower concentrations, amplification was stochastic. LAMP was unable to detect down to 195 copies/rxn in the presence of lysis buffer, indicating a loss in analytical sensitivity that was not observed with qPCR. Another difference between LAMP and qPCR is that although the LAMP TTP was delayed, the amplification rate and endpoint fluorescence in LAMP were not strongly affected (Fig. S7).

### Identifying a suitable TPW

**Figure 4:**
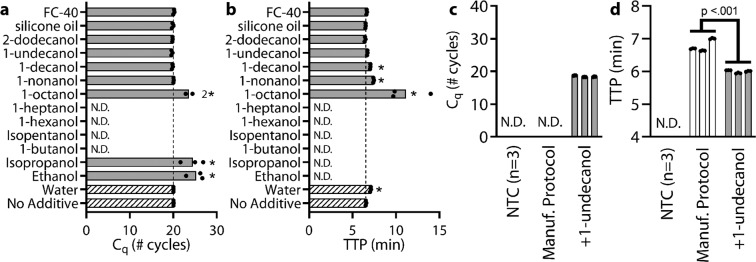
Identifying the most effective TPW in (**a**) qPCR and (**b**) LAMP reactions and subsequent validation of 1-undecanol as a candidate TPW with (**c**) qPCR and (**d**) LAMP at low eluent dilutions. TPW candidates for (**a**) qPCR and (**b**) LAMP reactions were spiked with 5 × 10^4^ copies λ phage DNA and primers, made to 10 µL, and 1 µL of each wash candidate was added to yield 11 µL total. The number 2 next to the 1-octanol bar indicates that only two of the three replicates amplified. The dashed lines show the C_q_ or TTP of the uninhibited 10 µL “No Additive” control. (**c**) qPCR with 2.2x diluted eluent and (**d**) LAMP with 2x diluted eluent on a λ phage DNA sample extracted with a Zymo Quick-Viral DNA/RNA kit. Protocol was performed according to manufacturer instructions as provided or with an additional TPW (+1-undecanol) between the ethanol wash and elution steps. Each bar represents the average of technical triplicates (black circles). We ran 6 extractions (3 silica columns x 2 conditions) and used the same eluent for both the qPCR and LAMP analyses. Samples marked N.D. were not detected within either 40 cycles or 40 min. NTC, no-template control. (**a**,**b**) We asked whether TPW candidates fell within the 99% CI of the “No Additive” control (qPCR: 20.01-20.17, LAMP: 6.25-6.83) with outliers indicated with a *. (**d**) We asked whether the average TTP was statistically different between the manufacturer protocol and the +1-undecanol condition using a *t*-test.

Next, we identified a suitable wash buffer that would reduce the carryover of extraction buffer and integrate easily into existing protocols. The ideal wash buffer would be added after the final ethanol wash but prior to the elution and it would have the following properties: (1a) it would be non-inhibitory or (1b) it would not transfer to downstream assays such as qPCR or LAMP, (2) it would remove previous washes from the column by an appropriate combination of solid-liquid and liquid-liquid interfacial properties and solubility of inhibitory components, and (3) it would not prematurely elute NAs from the column. We directly investigated criterion 1a by performing qPCR and LAMP reactions. We spiked reactions with *λ* phage DNA, diluted up to 10 µL, and we added an additional 1 µL of different wash buffer candidates to a total of 11 µL. As additional wash candidates, we tested increasing chain lengths of primary alcohols (or secondary alcohols if the primary form was unavailable), 5 centistokes (cSt) silicone oil, and FC-40 fluorocarbon oil (Fig. 4a,b). As an experimental control, we tested a “No Additive” condition, which was a 10 µL reaction with optimized reaction conditions and no inhibitors. To control for the effects of a 1 µL dilution on the reaction, we also tested a “Water” condition which was an 11 µL reaction with no inhibitors.

The “No Additive” control case showed a qPCR C_q_ of 20.09 ± 0.01 cycles (95% CI: 20.07–20.12) and a LAMP TTP of 6.54 ± 0.05 min (95% CI: 6.42–6.66). We note that 1 µL in 11 µL is a large fraction of the reaction mix (~9%), so we are overestimating buffer carry-over concentrations compared to normal operating conditions. The “Water” control showed no delay for qPCR and a 0.55 min delay for LAMP due to the dilution of LAMP reactants. For both qPCR and LAMP reactions, we found that long-chain alcohols with ≥9 chain lengths, silicone oil, and FC-40 were non-inhibitory for qPCR (within 1 cycle) and LAMP (within 1 min) compared to the “No Additive” condition (Fig. 4a,b). Octanol showed delays for qPCR (3.54 cycle difference) and LAMP (4.63 min difference), and only 2 out of 3 replicates amplified for qPCR. All alcohols with ≤8 chain lengths either had delayed amplification or the reaction was completely inhibited. Because long-chain alcohols, silicone oil, and FC-40 showed little to no inhibition of qPCR and LAMP, these candidates fulfilled criterion 1a.

These non-inhibitory wash candidates (long-chain alcohols, silicone oil, and FC-40), which we refer to as TPW, have low solubility in water (Table S7) and resulted in phase separation (Table S8). The TPW separates to either the top phase or the bottom phase (density dependent) while interacting minimally with the aqueous solution. As a result of reduced interactions with the aqueous solution, the TPW is less toxic to downstream reactions. In LAMP reactions with added alcohols (Fig. 4b), we also noticed that the TTP delay decreased as the solubility decreased (from 1-octanol to 2-dodecanol). The 1-octanol had the greatest delay (without completely inhibiting the reaction). We suspect that although 1-octanol mostly occupied its own phase, some 1-octanol dissolved in the aqueous phase and disrupted polymerase activity. Furthermore, we also noticed that the TTP for the very low solubility TPWs matched the “No Additive” condition rather than the “Water” condition, implying the reaction mix was not diluted by the 1 µL of added TPW.

Next, we evaluated criterion 1b (ensuring that the TPW does not transfer to qPCR and LAMP) as well as criterion 2 (the ability of the TPW to remove previous washes from the column) by running a NA extraction with or without TPW and adding the resulting eluent into qPCR and LAMP (Fig. 4c,d). Of our TPW candidates, we selected 1-undecanol for further evaluation because (i) it was non-inhibitory for qPCR and LAMP reactions and (ii) as an alcohol, 1-undecanol may function similarly to ethanol- or isopropanol-based washes. In these experiments (testing criteria 1b and 2), we first diluted a commercially purified *λ* phage DNA sample to 2.5 × 10^6^ copies and ran an NA extraction using the Zymo Quick-DNA/RNA Viral Kit. We either followed the manufacturer protocol or added an additional 300 µL 1-undecanol wash in between the Viral Wash Buffer and elution step. Using the manufacturer’s protocol, the resulting eluent is approximately 49 µL, but with the added TPW the resulting eluent is approximately 48 µL aqueous phase and ~1–2 µL 1-undecanol phase. Because we wanted to emphasize any potential inhibitory effects, we used a low dilution of eluent. For qPCR, we diluted 2.2x by adding 4.5 µL of eluent, 0.5 µL primers, and 5 µL qPCR reaction mix. For LAMP, we diluted 2x by adding 5 µL eluent, 0.5 µL primers, and 4.5 µL reaction mix. During the transfer of eluent into the reaction mix, we noticed that the phase separation yielded by the TPW resulted in minimal transfer of the TPW into downstream reactions (criterion 1b). The ~1–2 µL TPW separates from the aqueous phase and adheres to the walls of the tube, making it is easy to use a pipette to capture just the eluent.

Overall, we found that the addition of the 1-undecanol TPW greatly improved qPCR and LAMP performance at low dilution (Fig. 4c,d). Without the inclusion of the TPW, qPCR run at low dilution of eluent and following the manufacturer’s NA extraction protocol led to failed amplification in all 9 samples. However, with the TPW, the reaction completely recovered with a C_q_ of 18.46 ± 0.22 cycles. For LAMP and low dilution, we found that the manufacturer protocol amplified in 6.78 ± 0.17 min whereas our modified TPW protocol amplified in 6.00 ± 0.04 min (Fig. 4d). Not only was there a 0.78 min reduction in TTP (p < 0.01), variance was also reduced. Observing improvements for both qPCR and LAMP, we concluded there was reduced carryover of previous washes (criterion 2).

**Figure 5:**
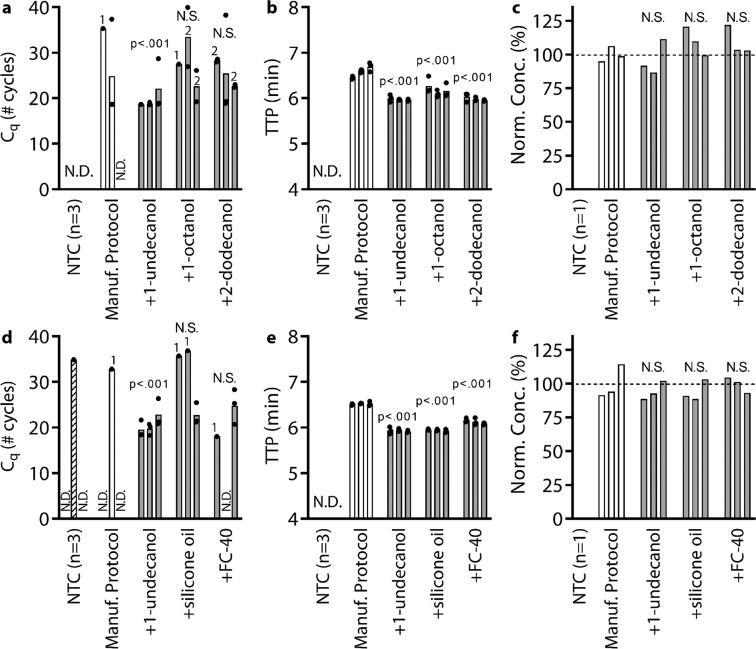
Comparing the performance of different TPWs with eluent at 2.2x dilution in qPCR (**a**,**d**), 2x dilution in LAMP (**b**,**e**), and 100x dilution in digital PCR (dPCR) (**c**,**f**). Samples were spiked with 2.5 × 10^6^ copies λ phage DNA and extracted in 50 µL water with a Zymo Quick-Viral DNA/RNA kit. We compared each manufacturer’s protocol (Manuf. protocol) with the same protocol plus an additional TPW of either 1-undecanol, 1-octanol, 2-dodecanol, silicone oil, or FC-40. To observe inhibition, a low eluent dilution was used in qPCR and LAMP with λ phage primers. To get a highly accurate quantification of NAs (for comparing these results), we ran each sample using dPCR with a high dilution of eluent (100x), which eliminates the effects of inhibitors. Each bar represents the average of qPCR or LAMP technical triplicates (black circles) or single dPCR measurements. We ran 24 extractions (3 silica columns x 8 conditions) and the same eluent was used to run the qPCR, LAMP, and dPCR analyses. Where shown, numbers above a bar indicate the number of samples which amplified if not all triplicates were detected. Dashed lines (panels c and f) indicate the average NA recovery following manufacturer protocol. Samples marked N.D. were not detected within 40 cycles by qPCR or 40 min by LAMP. (**a**–**f**) For each of the five TPW candidates, we asked whether the mean value was statistically different from the manufacturer protocol by *t*-test. N.S. stands for not significant (*P* > 0.05).

To confirm our result that the 1-undecanol TPW with low eluent dilutions led to significant improvements in qPCR and LAMP, we repeated this experiment twice more and found similar results. In total (Figs. 4 and 5), we ran 27 reactions (9 columns) following the manufacturer protocol and compared to 27 reactions (9 columns) with the added 1-undecanol wash. Each set of 3 columns showed a statistically significant (p < 0.01) difference comparing with and without 1-undecanol wash (p < 0.01) for qPCR and LAMP. For qPCR (triplicate) with the manufacturer protocol, we found 2/27 reaction wells with C_q_ between 18–22 cycles, 3/27 wells were delayed by 4 or more cycles, and 22/27 wells did not amplify. Of the 5 wells that amplified, the average C_q_ and standard deviation was 28.6 ± 9.2 cycles. Meanwhile, adding the 1-undecanol wash resulted in 25/27 wells with C_q_ between 18–22 cycles, 2/27 wells with a delayed C_q_, and all reactions amplified. The average C_q_ with the added 1-undecanol wash was 19.7 ± 2.5 cycles. We emphasize that in addition to more samples amplifying, we found that the C_q_ dropped and the measured variance among samples was reduced, thereby improving the accuracy, speed, and robustness of the diagnostic assay. For LAMP (triplicates), all 27 wells with TPW (10.23 ± 0.06 min) had a faster TTP than all 27 wells following manufacturer protocols (11.36 ± 0.27 min). Again, we find that the 1-undecanol wash improved the speed and robustness (reduced variance) of the assay.

Next, we investigated whether this result was specific to 1-undecanol or TPWs in general (Fig. 5a,b,d,e). For this experiment, we chose 2-dodecanol because it is the longest chain alcohol we tested and 1-octanol because it is the shortest chain alcohol for which both qPCR and LAMP still amplified (Fig. 4a,b). We expect 2-dodecanol to perform similarly to 1-undecanol because they are compositionally similar and both were previously found to be non-inhibitory for qPCR and LAMP (Fig. 4a,b). Accordingly, we expect 1-octanol might perform worse than the other TPW candidates, given its higher solubility and previously observed delays. We also chose silicone oil and FC-40 to evaluate nonalcoholic forms of TPW. The result of our study found that all five TPW candidates outperformed the manufacturer protocol. In qPCR reactions, 7/9 reactions amplified with 2-dodecanol wash, 5/9 for 1-octanol, 5/9 for silicone oil, and 4/9 for FC-40 whereas without the TPW (following the manufacturer protocol) amplification often failed (5/27). For LAMP, all TPWs conditions amplified with a faster TTP than manufacturer protocol. (*P* < 0.01).

We hypothesize 1-undecanol and 2-dodecanol performed best (greatest number of successfully amplified qPCR reactions and faster LAMP TTPs) because these two TPW candidates met all of our criteria (1a. non-inhibitory, 1b. low transfer to downstream assays, 2. remove previous wash, and 3. do not elute NAs). Meanwhile, we hypothesize 1-octanol performs slightly worse because 1-octanol is inhibitory to qPCR and LAMP (criterion 1a). However, these inhibitory effects are minimal because 1-octanol phase-separated from the eluent and, as a result, only a small volume of 1-octanol was carried-over into the downstream reactions (criterion 1b). Lastly, we observed that both silicone oil and FC-40 demonstrated slightly worse performance than the other TPW candidates. A potential explanation for the lower performance of silicone oil and FC-40 is that during the TPW step, the alcohols mixed with the previous ethanol-based wash whereas silicone oil and FC-40 did not (Table S8). As a result, this allows the alcohol-based TPWs to dilute and more effectively cleanse droplets of ethanol trapped in the column (criterion 2).

Next, we evaluated whether or not the TPW meets criterion 3 (NAs are effectively eluted from the column during the TPW or lost due to premature elution or incomplete elution) (Fig. 5c,f). For this experiment, we used a 100x dilution to reduce buffer concentrations to non-inhibitory levels followed by digital PCR (dPCR); dPCR is a highly sensitive method for quantifying NAs that detects the same target (same primers) as qPCR. Although triplicates are commonly tested for qPCR and LAMP, for dPCR experiments we ran duplicates measurements each with more than 15,000 individual reactions. We merged the results from both experiments and used the Poisson distribution to calculate the final concentration using Bio-Rad’s QuantaSoft analysis software. We normalized all dPCR concentrations to the average concentration of the three extractions following the manufacturer protocols. We found that the TPW did not appreciably affect the NA recovery, fulfilling our final criterion (3) for an ideal wash buffer. Furthermore, all highly diluted dPCR measurements showed similar NA recovery between manufacturer protocol and TPW conditions, whereas low dilutions resulted in stark differences for both qPCR and LAMP, further confirming that inhibitors are responsible for delays in C_q_ and TTP.

### TPW validation for different kits with high and low dilution

To evaluate the generality of our approach and better understand the mechanism, we tested three extraction kit protocols with and without the added TPW. We also wanted to evaluate whether there is a difference in downstream amplification between high eluent dilution (10x) and low eluent dilution (2x or 2.5x). We evaluated Zymo’s kit D7021 using either the newer protocol (Zymo Quick-DNA/RNA Viral Kit) or the older protocol (Zymo ZR Viral DNA/RNA Kit). Although both protocols use the same buffers, the Zymo Quick Kit has three wash steps (two viral wash buffers and one ethanol wash) whereas the Zymo ZR kit has one viral wash buffer step. By default, the Zymo kits do not include a “dry spin.” The Qiagen QIAquick uses a different set of buffers, has one wash step, and by default includes a “dry spin.” In this experiment, all kits extractions were performed on pure water (there are no NAs during the extraction, Fig. S1) to ensure we are only evaluating the effects of buffer inhibitors. The subsequent qPCR and LAMP reactions were then spiked with 5 × 10^4^ *λ* DNA copies. As a control, water was added to qPCR or LAMP (rather than kit extract) to represent the best-case reaction without inhibitors (“No Extract”).

**Figure 6:**
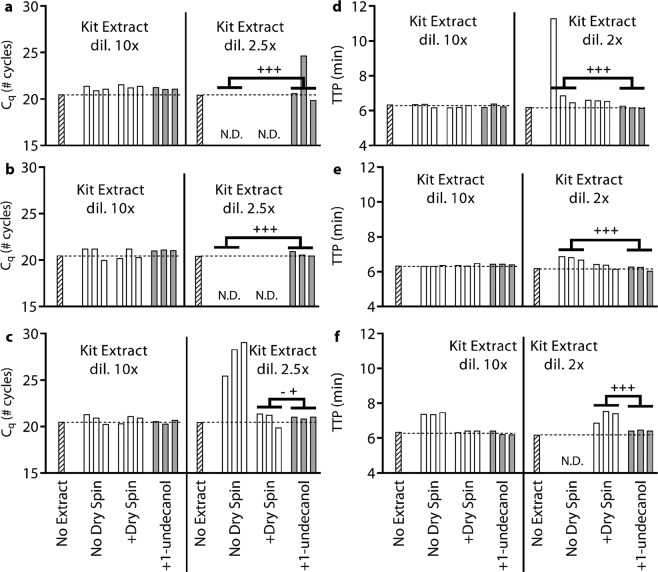
Evaluation of TPW for different silica-column NA extraction kit protocols on pure water samples using (**a**–**c**) qPCR and (**d**–**f**) LAMP. All reactions were spiked with 5 × 10^4^ copies λ phage DNA and primers. By manufacturer protocol, the (**a**,**d**) Zymo Quick-DNA/RNA Viral Kit and (**b**,**e**) Zymo ZR Viral DNA/RNA Kit do not include the dry spin (+dry spin) whereas the (**c**,**f**) Qiagen QIAquick PCR Purification Kit does. The left of each graph shows high dilution and the right shows low dilution. Each bar represents the result from a single qPCR or LAMP measurement. We ran 27 silica-column extractions (3 silica columns × 3 conditions × 3 extraction protocols) and the kit extract was shared between high and low dilutions of both qPCR and LAMP. Dashed lines show the C_q_ or TTP for a reaction without inhibitors (“No Extract”). Samples marked N.D. were not detected within either 40 cycles or 40 min. (**a**–**f**) We asked whether the manufacturer protocol replicates (“No Dry Spin for Zymo kits, “+dry spin” for Qiagen kit) fell within the 95% CI of the corresponding +1-undecanol condition for the low kit extract dilution case. The number of replicates that lie outside the 95% CI are indicated by the number of + (above) and - (below).

We did not observe inhibition at 10x dilution following manufacturer protocols (Fig. 6), which confirmed that the standard 10x or more dilution into qPCR and LAMP prevents the inhibitory effects we see at lower dilutions. With a 10x dilution, we noticed that the “No Dry Spin” condition using the Qiagen kit with LAMP resulted in ~1 min delay. We note that the Qiagen kit manufacturer protocol requires the dry spin. Without the dry spin, we noticed the Qiagen kit extract had substantially more volume (~65 µL) than when the dry spin was included (~49 µL). This implies ~16 µL (25%) carryover of Buffer PE into the kit extract. The volume of kit extract from Zymo kits, however, was not noticeably affected by the addition of the dry spin (~49 µL with or ~49 µL without).

However, when we used 2x or 2.5x dilutions we observed significant inhibition (Fig. 6). With the Zymo kits and qPCR, there was no amplification whether or not an additional dry spin was added (Fig. 6a,b), contradicting Zymo’s “no buffer contamination” claim. For the Qiagen kit (Fig. 6c) and qPCR, the dry spin performs quite well, matching the No Extract control. With the Zymo kits and LAMP (Fig. 6d,e), there are delays when following the protocol (no dry spin) but this is slightly improved by adding a dry spin. With the Qiagen kit and LAMP (Fig. 6f), we observe total reaction inhibition without the dry spin and a 1.1 min delay following the manufacturer protocol. In summary, these results prove that inhibitors are carried into the elution, the additional dry step is helpful for removing wash buffers, and high dilution is the responsible for reducing concentrations to non-inhibitory levels.

Lastly, we used our modified protocol utilizing 1-undecanol TPW and found substantially improved performance, even at low dilutions of the kit extract. We calculated the 95% confidence interval (C.I.) for each 1-undecanol condition at the low dilution and counted the number of outliers when following the manufacturer protocol. For all kits and combinations, we find that the TPW matches performance (Qiagen qPCR) or substantially improved performance (Zymo ZR and Zymo Quick qPCR, all LAMP conditions). The most drastic improvement is for the Zymo ZR kit and qPCR, which failed to amplify with the manufacturer protocol but completely recovered when we added the TPW (Fig. 1c is a subset of Fig. 6b showing “No Dry Spin” and “+1-undecanol”). Given the dramatic improvements and ease of adding the TPW, we recommend silica-column kit manufacturers further evaluate the TPW and consider inclusion with their kits.

We evaluated whether in some cases the TPW could be considered as an alternative for ethanol-based washes (Fig. S8). As a comparison, we used the Zymo ZR kit which only has one wash step (viral wash buffer). We either replaced the viral wash-buffer step with a dry spin (control), ethanol (control), or different TPW solutions. Briefly, we found that at least under these clean conditions, ethanol wash slightly outperforms the viral wash buffer, long-chain alcohol washes have the best performance, and non-alcohol washes (silicone oil and fluorocarbon oil) led to failed amplifications.

### TPW validation for different reaction mixes with high and low dilution

To understand how different reaction mixes respond to buffer carry-over, we compared NEB’s SsoFast mix to NEB’s Luna mix and our manually prepared LAMP mix to NEB’s pre-made LAMP mix. Using a Zymo Quick-DNA/RNA Viral Kit for extractions, we found that the Luna mix amplified at a 2.2x dilution of kit eluent whereas the SsoFast mix did not (Fig. S6a,b). This result implies that the Luna kit is more tolerant to the Zymo extraction buffer inhibitors than to those in the SsoFast mix. When we compared experiments with and without the TPW, we again observed that the inclusion of the TPW improved downstream assay performance, recovering amplification for the SsoFast mix and reducing the C_q_ from 19.1 to 18.4 cycles for the Luna qPCR assay. The manually prepared LAMP mix performed similarly to the pre-made LAMP kit, and again the TPW improved performance at low eluent dilution (2.86x). The TTP for the home-made mix was reduced from 7.4 to 7.0 min and the TTP for the pre-made mix was reduced from 7.9 to 7.4 min (Fig. S6c,d).

### TPW validation for magnetic-bead extractions

We next tested whether TPW would improve magnetic bead extractions. Sur *et al*. previously found that transferring magnetic particles through a hydrophobic liquid effectively reduced PCR inhibitors^51^. This method, termed immiscible phase filter (IPF), allowed for the replacement of multiple wash steps with a single pass through an immiscible liquid. At a 5x dilution of eluent into RT-qPCR, the IPF method showed no statistical difference in detected copies compared to commercial kits for HIV-1 spiked into plasma, Chlamydia and Gonorrhea spiked into urine, and proviral HIV-1 DNA integrated with peripheral blood mononuclear cells in whole blood. Another previous study conducted by Berry *et al*. described the IFAST (immiscible filtration assisted by surface tension) device^52^, and further analyzed their method by examining surface tensions and energies associated with the aqueous phase, immiscible phase, and their device material. The IFAST device reduced total NA extraction operation time to less than 5 min while showing similar performance to commercial extraction kits with operation times between 15 to 45 min (eluent dilution unspecified).

**Figure 7:**
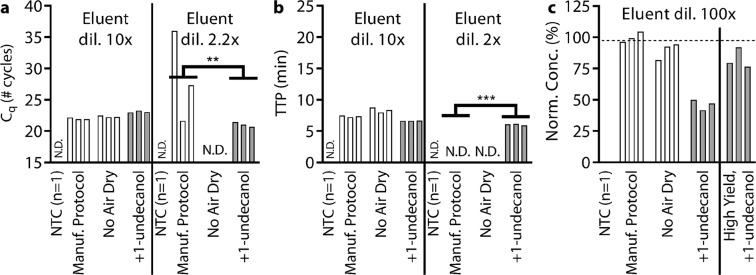
Evaluating TPW for compatibility with Zymo Quick-DNA/RNA MagBead extraction with (**a**) qPCR, (**b**) LAMP, and (**c**) dPCR. Extraction performed on 1 × 10^6^ λ phage DNA copies with either a 10 min air dry (Manuf. protocol), no air dry, or with the air dry replaced by a TPW (+1-undecanol) step. The resulting eluent is spiked at either high dilution or low dilution into (**a**) qPCR and (**b**) LAMP or 100x dilution into (**c**) dPCR. For dPCR (**d**), the bars to the right of the solid black line show the results for an extraction protocol with a +1-undecanol wash using a high-yield protocol from a separate experiment (normalized to the no TPW control in that experiment). Bars represent single qPCR and LAMP or the merged result from a duplicate dPCR measurement. Dashed line in dPCR (**c**) indicates the average NA recovery following manufacturer protocol. We ran 9 extractions (3 magnetic-bead extractions x 3 conditions) and the eluent was shared among qPCR, LAMP, and dPCR analyses. Samples marked N.D. were not detected within either 40 cycles for qPCR or 40 min for LAMP. (**a**,**b**) We asked whether the manufacturer protocol replicates fell within the 95% CI of the corresponding +1-undecanol condition for the low eluent dilution case. The number of replicates that lie outside the 95% CI were indicated by the number of *s.

Here with test the TPW with a commercial magnetic bead extraction kit and evaluate both high and low dilution of eluent into LAMP and qPCR. A schematic of the magnetic-bead protocol is shown in Fig. 1b. Using a Zymo Quick-DNA/RNA MagBead Extraction kit, we started with 1 × 10^6^ copies *λ* DNA and eluted with 50 µL. By default, the protocol requires a 10 min air dry step to allow residual ethanol from the wash step to evaporate. We tested the manufacturer protocol, protocol without the air dry step, and the protocol where the air dry step was replaced with a 1-undecanol TPW. At 10x dilution into qPCR (Fig. 7a), omitting the dry step has no effect. Adding the 1-undecanol TPW led to a 1.1 cycle delay, which corresponds to a decrease in NA extraction efficiency (Fig. 7c) rather than an inhibitory delay. At 10x dilution into LAMP (Fig. 7b), omitting the air dry step causes a 1 min delay, and including the TPW leads to a 0.7 min TTP improvement. At low dilutions, the inhibitory effects are more drastic, and the TPW clearly outperformed the kit protocol with 2 of 3 manufacturer protocol samples performing worse by qPCR and 3 of 3 manufacturer protocol non-detects.

Further experimentation with the MagBead kit revealed that the greater the volume of 1-undecanol carryover, the lower NA recovery we observed. In the experiment shown (Fig. 7), the three extractions had approximately 30 µL, 24 µL, and 22 µL of 1-undecanol carryover as measured by pipette. We found that following the initial 1-undecanol aspiration, a significant volume of 1-undecanol remains stuck to the magnetic beads and walls of the tube. To improve NA yield, we developed a modified protocol in which we aspirate the 1-undecanol, wait at least 1 min, and aspirate any remaining 1-undecanol that slid down the tube due to gravity. This modification led to high yield of NAs after TPW for 1-undecanol (Fig. 7c) and for other compounds (Fig. S9).

### TPW validation for RT

**Figure 8:**
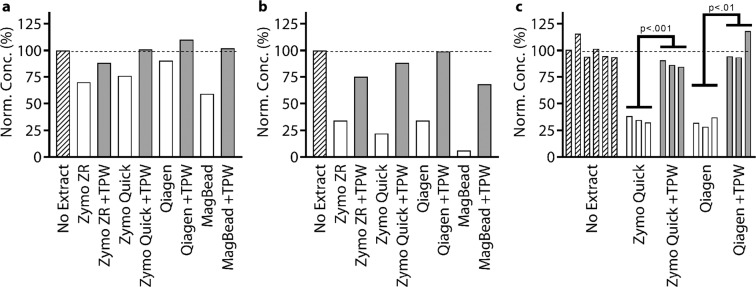
Measurement of reverse transcription (RT) efficiency on *Neisseria gonorrhoeae* RNA using 16S rRNA gene primers with (**a**) 10x dilution or (**b**,**c**) 2x dilution of extractions from different commercial kits into RT reaction mix. NA concentration quantified by digital PCR after 100x dilution of post-transcribed RT mix. (**c**) We asked whether RT yield comparing with and without TPW was statistically different using a *t*-test.

We next tested how extraction buffer carryover and TPW would affect RT. For applications requiring high sensitivity, the starting sample might only contain a few cells. In these scenarios, it is beneficial to detect RNA because many RNA copies can be made from a single DNA copy. To evaluate whether or not buffer carryover affects RT, we ran an RT experiment using RNA from *N. gonorrhoeae*, a pathogen with clinical and diagnostic relevance (Fig. 8). First, a high concentration of RNA was extracted using a Zymo ZR Viral DNA/RNA Kit, and the extracted RNA was diluted 100-fold to reduce the concentration of inhibitors. Separately, we ran kit extractions on pure water samples for all previously examined NA extraction kits. We combined RNA with kit extractions into RT reactions containing WarmStart Rtx, NG 16S rRNA PCR primers, and other reaction components. We emphasize that all reactions contained equal concentrations of RNA, and were expected to produce equal levels of DNA. In each RT reaction, we either added 1 µL kit extract to 9 µL reaction mix (10x) or 5 µL kit extract to 5 µL RT reaction mix (2x). For the “No Extract” condition, we added either 1 µL or 5 µL water. Following RT, the transcribed DNA was then diluted an additional 100x and added to dPCR mix (reaction mix, PCR primers) for quantitative analysis. By separating the RT reaction and quantification with dPCR, we can clearly investigate the effects of buffer inhibition on RT alone (whereas with a 1-step RT-dPCR reaction it is difficult to determine whether inhibition affects RT or dPCR). We observed a clear trend: using kit extracts while following manufacturer protocols led to a reduction in the amount of DNA that was transcribed. This trend was observed even at a 10x dilution of kit extract into the RT reaction, implying that RT is more strongly inhibited than qPCR or LAMP (Fig. 8a). However, when the TPW was added to the NA extraction kit, transcription efficiency was improved for all kits. These trends are even more pronounced when examining a 2x dilution of kit extract into the RT reaction (Fig. 8b). These results were further confirmed with greater sample size in a separate experiment for 2x dilution of kit extract into RT reaction (Fig. 8c). We found that the TPW significantly improved the efficiency of the RT reaction.

## Conclusions

In this manuscript, we evaluated how the buffers from solid-phase silica-column centrifugation and magnetic-bead extraction kits are carried over into the eluent and inhibit downstream amplification reactions. Using kits from leading manufacturers, we repeatedly observed that as expected, a high (10x) dilution of eluent showed little to no inhibition of qPCR or LAMP reactions. However, carried-over extraction buffers caused delays or completely inhibited amplification and reverse transcription at low (2–2.5x) dilutions of eluent. We observed reaction inhibition using two different silica-column centrifugation kits (3 protocols: Zymo ZR, Zymo Quick, Qiagen QIAquick) and a magnetic-bead kit (Zymo MagBead) when using the manufacturer protocols.

We reduced the inhibition due to carryover by developing a TPW protocol that improved eluent purity and led to more efficient and reproducible reactions. We showed that the inclusion of a dry spin step, although helpful, still generated buffer carryover which inhibited qPCR and LAMP at low eluent dilutions. We discovered that the inclusion of a TPW step greatly reduced buffer carryover, and we found that low solubility compounds exhibited the best performance. Using the TPW protocol improved eluent purity, leading to more efficient (reduced delays in C_q_ or TTP) reactions. The addition of the TPW also improved the efficiency of RT reactions.

Furthermore, TPW improved reproducibility of amplification reactions by reducing C_q_ and TTP variations between measurements (Fig. 7a at 2.2x dilution), and at low target concentrations leading to more repeatable detection (Fig. 7b, 2x dilution). Reproducibility is an important aspect of nucleic-acid assays in biological research and diagnostic assays. Given the high degree of sensitivity of reactions to levels of carryover (Fig. 2), especially at low target NA concentrations (Fig. 3), it is expected that slight variation in the extent of carryover can lead to high variation in the performance of a NA assay. High purity eluent from TPW was compatible with low dilutions into amplification mix, improving assay sensitivity because more NAs could be added to each reaction.

We anticipate the addition of the TPW would improve NA extraction purity and performance of downstream assays in a variety of applications. We have demonstrated performance of TPW for a range of commercial extractions kits and a range of nucleic-acid targets. One limitation of this study is that it is not exhaustive: we have not tested every possible kit, every possible sample type, every possible NA reaction, and every possible nucleic-acid target. However, TPW is inexpensive and easy to incorporate into both silica-column (one additional spin) and magnetic-bead extractions (one additional aspiration), and therefore we encourage researchers and commercial suppliers to test TPW in their specific workflows and protocols. In particular, we expect to use the TPW extraction in combination with lyophilized reagents, which requires no dilution, and is highly desirable for point-of-care diagnostics. Finally, the TPW will enable the field to develop new methods of sample preparation, such as pressure- or vacuum-based NA extractions, that are simpler, quicker, and more portable than current protocols.

In addition to reducing extraction buffer carryover, we hypothesize the TPW could also reduce carryover of some compounds originating from the sample by removing them from the solid phase. For example, long-chain alcohols might remove nonpolar compounds better than traditional wash buffers (ethanol or isopropanol). This hypothesis remains to be tested in future work. Furthermore, we anticipate that improved eluent purity from the added TPW would enable high-sensitivity analyses that were previously difficult or impossible because high dilution of eluent has been the *de facto* standard. Improved eluent purity would be especially valuable for more challenging reactions, including long amplicons (DNA and RNA), targets with high GC content, and highly structured or chemically modified RNA targets (e.g. rRNA, tRNA). By enabling the use of lower dilutions, this method would enhance performance of NA analysis in applications where sensitivity and reproducibility are critical, including single-cell sequencing, cell-free circulating DNA analyses and SNP detection, and molecular diagnostics.

## Supplementary information

Supplementary Information.

## Data Availability

Full dataset available through CaltechDATA, 10.22002/D1.1298; https://data.caltech.edu/records/1298.
