# Supplementary material for: Two-phase wash to solve the ubiquitous contaminant-carryover problem in commercial nucleic-acid extraction kits
Source: Sci Rep. 2020 Feb 6;10:1940. doi: 10.1038/s41598-020-58586-3 (PMC7004994; doi:10.1038/s41598-020-58586-3)
Supplement: Supplementary file 1 — Supplementary Information. [file 41598_2020_58586_MOESM1_ESM.pdf]

Supplementary Information for:

# Two-phase wash to solve the ubiquitous contaminant-carryover problem in commercial nucleic-acid extraction kits

Erik Jue,[a] Daan Witters,[b] and Rustem F. Ismagilov [a,b]

[a]Division of Biology and Biological Engineering

[b]Division of Chemistry and Chemical Engineering

California Institute of Technology 1200 E. California Blvd., Pasadena, CA, 91125 United States

* Correspondence to: rustem.admin@caltech.edu

**Contents**:

*Kit extractions on "pure water"*

Figure S1

*Full data set for buffer inhibitors in qPCR and LAMP*

Figures S2-S5

*Buffer inhibitors in qPCR and LAMP*

*Tables S1-S4*

*TPW validation for different reaction mixes with high and low dilution*

Figure S6

*Inhibitory effects on NA amplification curves*

Figure S7

*TPW screen with qPCR and LAMP*

Tables S5-S6

*Solubility table and ethanol phase separation for TPW candidates*

Table S7

*Evaluating a 3-step centrifugation extraction with TPW*

Figure S8

*Evaluating a low-carryover, high-yield MagBead protocol*

Figure S9

*Statistical analysis methods*

*References*

*Kit extractions on “pure water”*

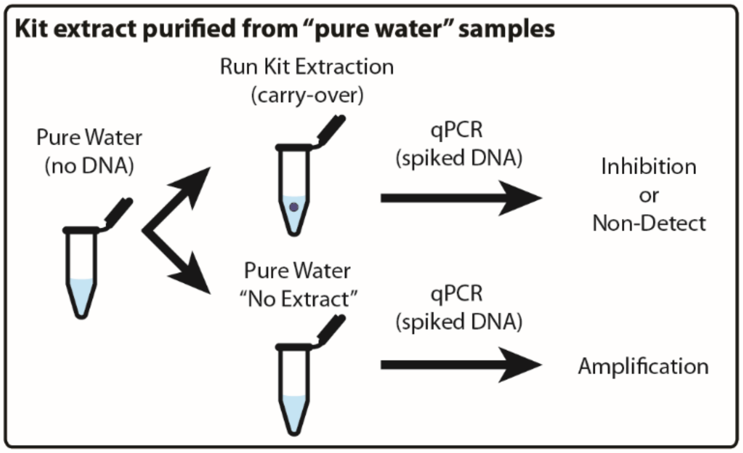


**Figure S1. Example protocol used for experiments performed on “pure water.”**

Typically, it makes sense to run controls with nucleic acids (NAs) spiked into the sample prior to the NA extraction step. However, in our “pure water” experiments we wanted to observe the effects of buffer carry-over independently of NA yield. We subsequently ran NA extractions on “pure water” samples to obtain eluent containing buffer carry-over (kit extract). We then used the original “pure water” sample as the non-inhibited control and compared to the kit extract (elution from kit extraction performed on pure water) in NA spiked downstream reactions. This approach was used to generate Figs. 1c, 6, and 8.

*Full data set for buffer inhibitors in qPCR and LAMP*

Figs. S2-5 show the full data set for buffer dilutions in qPCR and LAMP. The A-C panels of each figure (providing $C_q$ and TTP data) were presented in the main text. Changes in the endpoint RFU were highly concordant with changes in $C_q$ or TTP. The melting-temperature (Tm) effects showed up at low concentrations of inhibitors, suggesting that Tm can be an effective indicator for the presence or absence of inhibitors in sample.

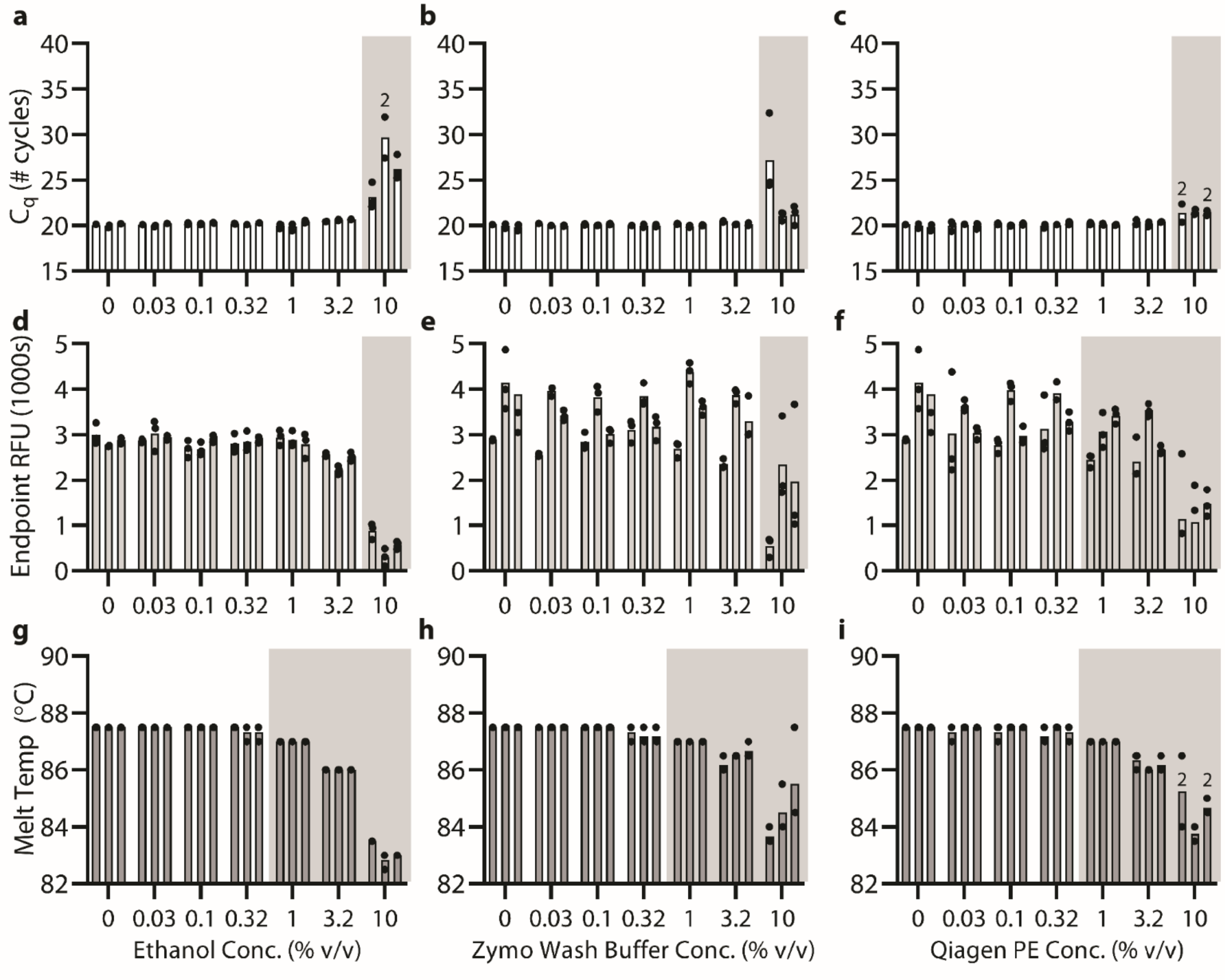


**Figure S2. (a-c) $C_q$, (d-f) endpoint fluorescence, and (g-i) melting temperature for qPCR on 5 x $10^4$ λ phage DNA copies in the presence of ethanol, Zymo Viral Wash Buffer, or Qiagen PE Buffer.** Gray background indicates an average $C_q$ delay of at least 0.5 cycles, RFU decrease of at least 500 RFU, or a melting temperature change of at least 0.5 °C compared with the 0% buffer condition.

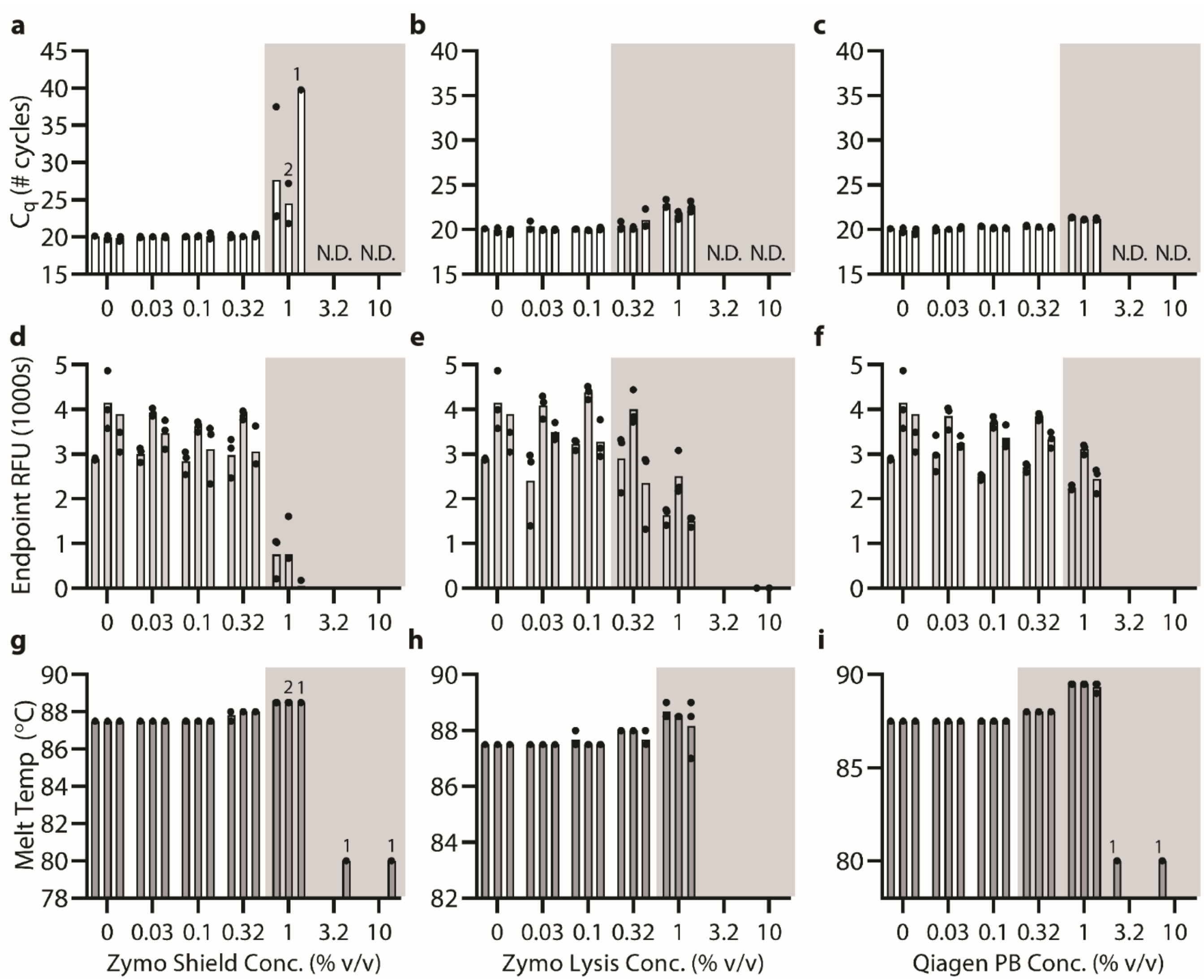


**Figure S3. (a-c) $C_q$, (d-f) endpoint fluorescence, and (g-i) melting temperature for qPCR on $5 \times 10^4$ λ phage DNA copies in the presence of Zymo DNA/RNA Shield, Zymo Viral DNA/RNA Buffer, or Qiagen PB Buffer.** Gray background indicates an average $C_q$ delay of at least 0.5 cycles, RFU decrease of at least 500 RFU, or melting temperature change of at least 0.5 °C compared with the 0% buffer condition.

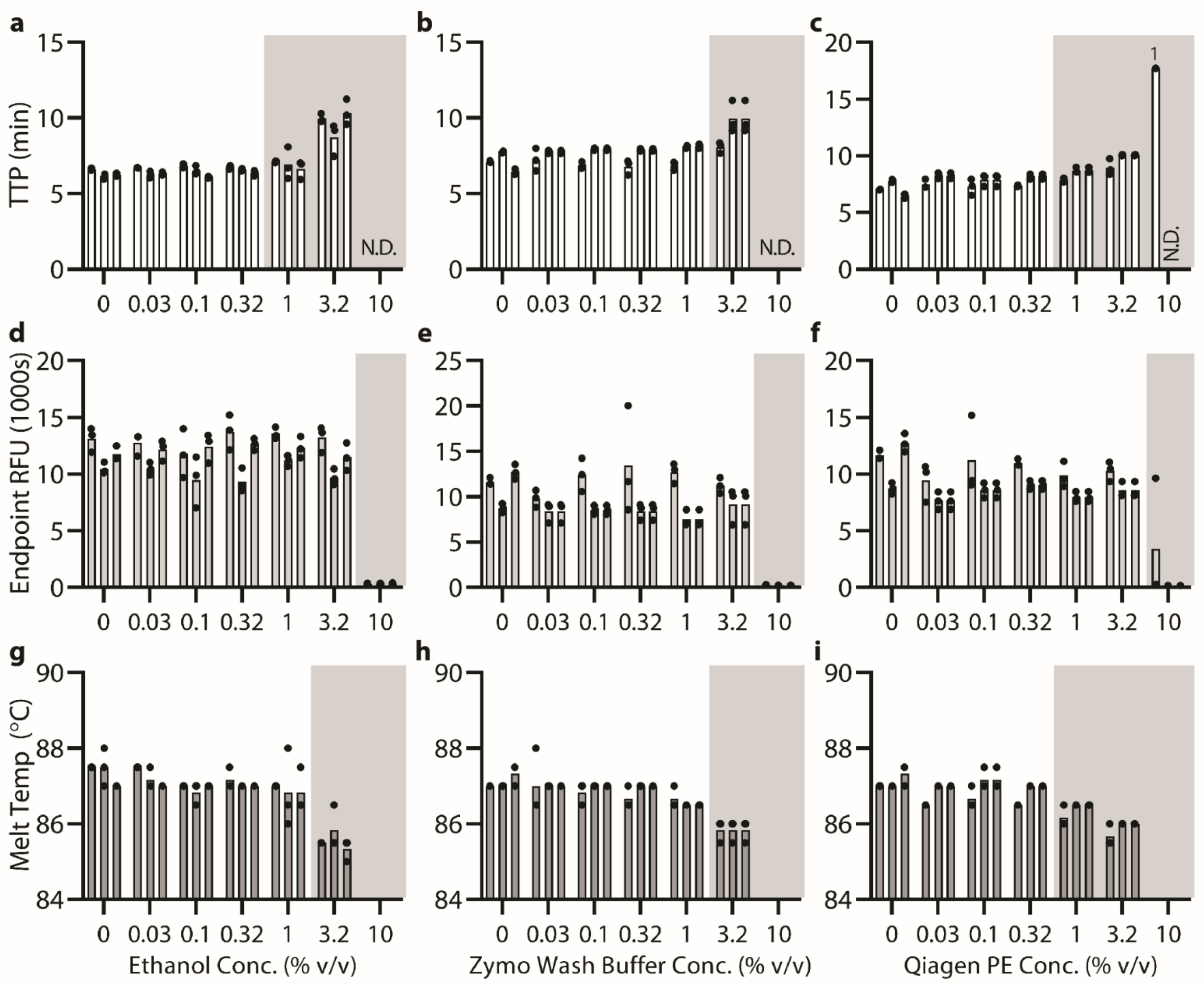


**Figure S4. (a-c) TTP, (d-f) endpoint fluorescence, and (g-i) melting temperature for LAMP on 5 x $10^4$ λ phage DNA copies in the presence of ethanol, Zymo Viral Wash Buffer, or Qiagen PE Buffer.** Gray background indicates an average TTP delay of at least 0.5 min, RFU decrease of at least 5000 RFU, or melting temperature change of at least 0.5 °C compared with the 0% buffer condition.

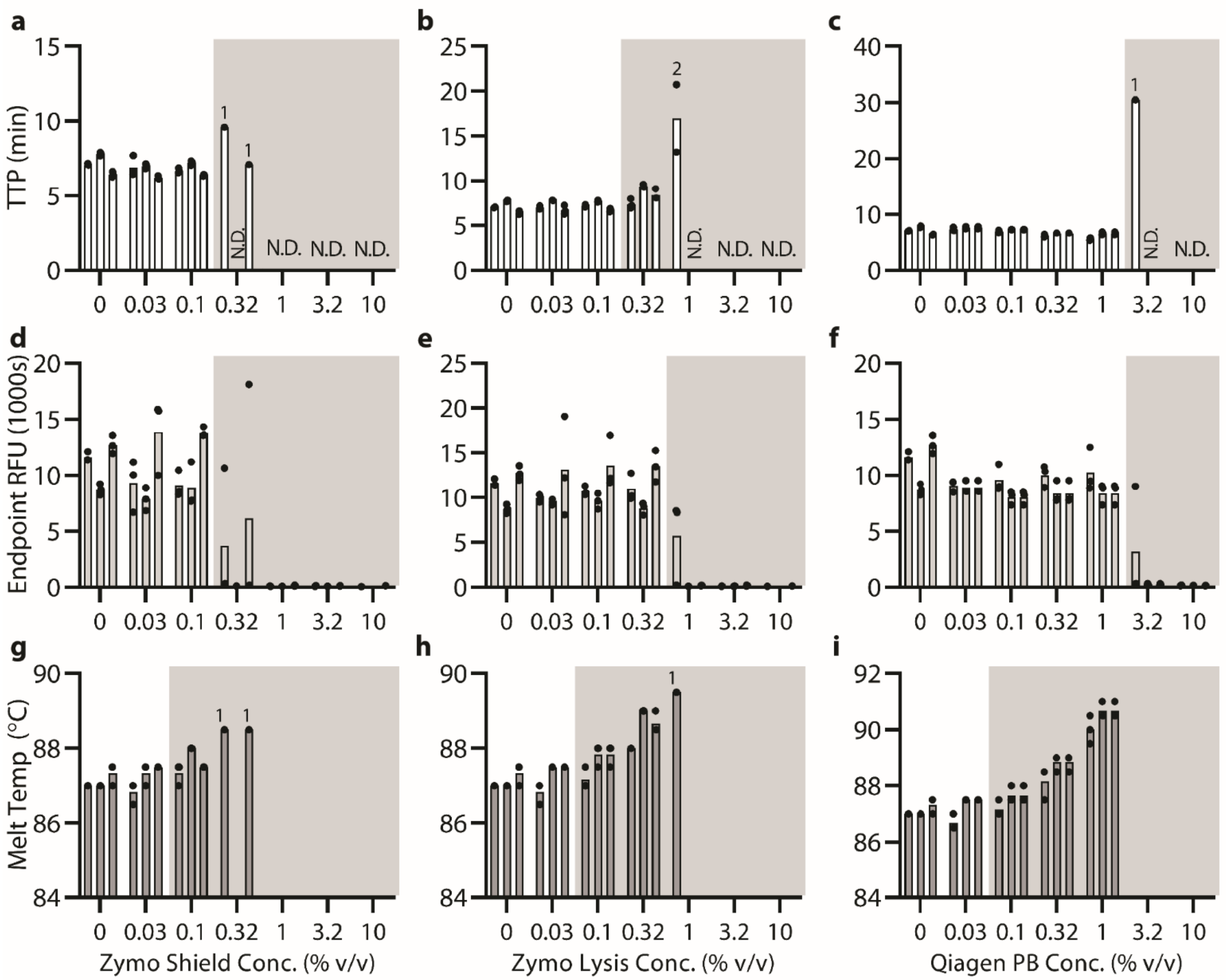


**Figure S5. (a-c) TTP, (d-f) endpoint fluorescence, and (g-i) melting temperature for LAMP on 5 x $10^4$ λ phage DNA copies in the presence of Zymo DNA/RNA Shield, Zymo Viral DNA/RNA Buffer, or Qiagen PB Buffer.** Gray background indicates an average TTP delay of at least 0.5 min, RFU decrease of at least 5000 RFU, or melting temperature change of at least 0.5 °C compared with the 0% buffer condition.

*TPW validation for different reaction mixes with high and low dilution*

We compared NEB's SsoFast EvaGreen Supermix to NEB's Luna Universal qPCR master mix and a manually prepared LAMP mix to NEB's pre-made WarmStart LAMP Kit. For the SsoFast mix, we used 500 nM primers (NEB recommended 300-500 nM) and for the Luna mix we used 250 nM primers (NEB recommendation). The same primer concentration was used for the manually prepared LAMP mix and NEB's pre-made mix. For the LAMP comparison, the lowest possible dilution was 2.86x because NEB's pre-made LAMP mix required 65% of the reaction volume (WarmStart LAMP 2X master mix, 50x fluorescent dye, primers).

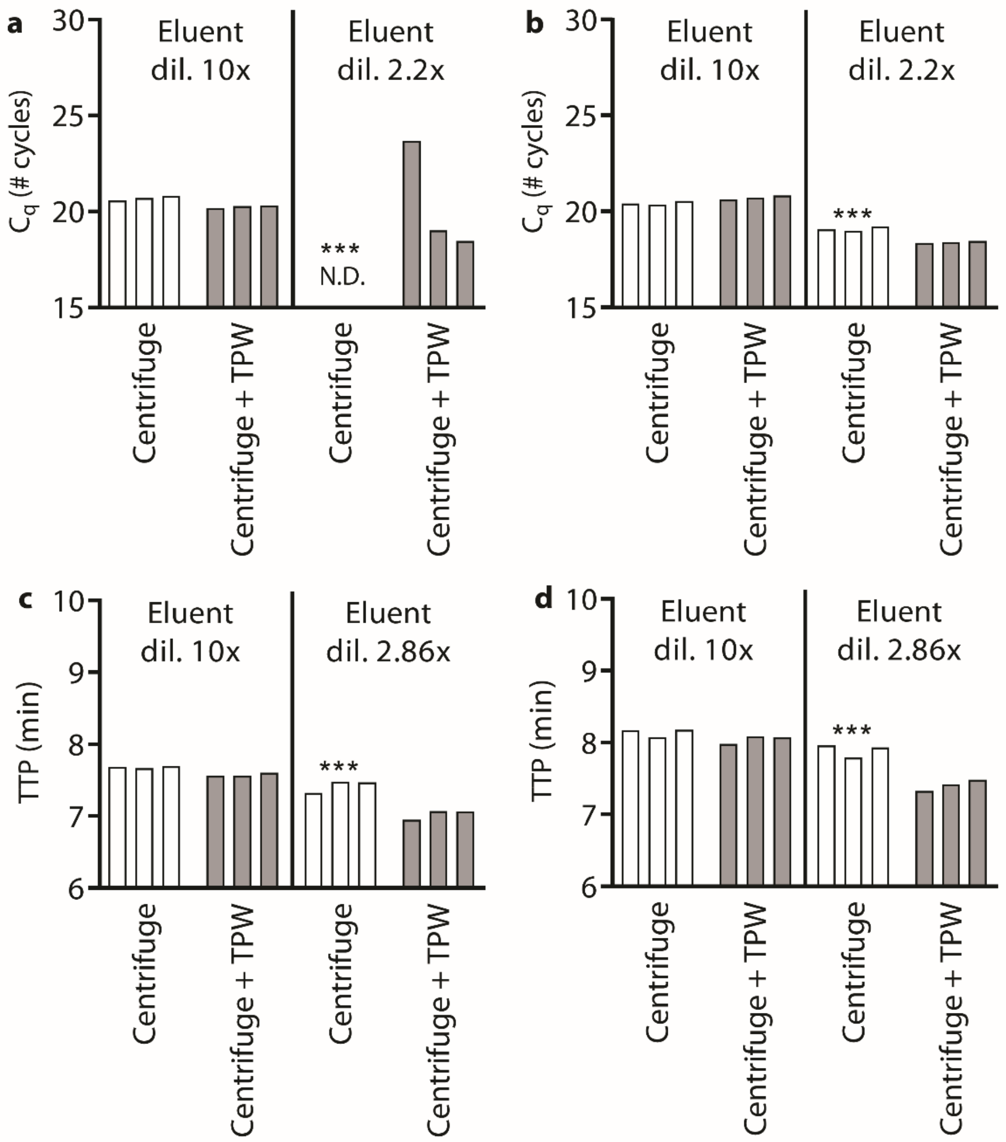


**Figure S6. Evaluation of extraction buffer inhibition on different assays and improvements due to the addition of a TPW.** We compared the (a) NEB SsoFast mix to the (b) NEB Luna mix and we compared a (c) manually prepared LAMP mix to an (d) NEB pre-made LAMP mix. Kit eluent was obtained by performing a Zymo Quick-DNA/RNA Viral Kit on 2.5 x $10^5$ copies λ phage DNA and eluting with 50 µL water. The left side of each graph shows high dilution and the right side shows low dilution. We ran six silica-column extractions in total and the same kit extract was shared among the high and low dilutions of all assays. Samples marked N.D." indicate not detected within either 40 cycles (qPCR) or 40 min (LAMP). All negative controls were clean (not shown). For the low eluent dilution conditions, we asked how many replicates following the standard centrifugation protocol fell outside of the 95% confidence interval for the corresponding centrifuge +TPW condition (indicated by number of *).

*Buffer inhibitors in qPCR and LAMP*

We note that 3.2% Qiagen PE Buffer in LAMP caused a large delay (6.0 min ΔTTP), but this difference does not measure as statistically significant by t-test. This is due to a bias introduced by a single non-detect (8 out of 9 amplified) which greatly increased the measured standard deviation. If we exclude the non-detect from the analysis (rather than assigning the non-detect to a value of 46.7 min), the t-test measures a *P*-value of 0.002. Also of potential interest, Qiagen PB Buffer appears to have sped up LAMP at low concentrations (0.1% - 1%). This result is unexpected, and further testing is required to validate this surprising result, which we hypothesize is not generalizable (e.g. could be primer or reaction mix dependent).

**Table S1. Summary of ethanol-based buffer dilutions for qPCR.** The average and standard deviation were calculated from 9 replicates. The $\Delta C_q$ is calculated by subtracting the average value for a given buffer concentration from the water condition (0%). A positive value indicates a cycle delay when adding the buffer. *P*-values were calculated by a 1-tailed unequal variance t-test compared to the water condition (0%). A * indicates a delay of at least 0.5 cycles and *P*-value <0.05. Non-detects were assigned a value of 40 cycles. VWB = Zymo Viral Wash Buffer; PE = Qiagen PE Buffer.

| | Ethanol | | | | | VWB | | | | | PE | | | | |
|---|---|---|---|---|---|---|---|---|---|---|---|---|---|---|---|
| | Avg | Std | ΔCq | p | * | Avg | Std | ΔCq | p | * | Avg | Std | ΔCq | p | * |
| 0% | 20.1 | 0.2 | | | | 20.0 | 0.3 | | | | 20.0 | 0.3 | | | |
| 0.03% | 20.1 | 0.1 | 0.0 | 0.499 | | 20.1 | 0.1 | 0.1 | 0.171 | | 20.0 | 0.3 | 0.0 | 0.431 | |
| 0.1% | 20.2 | 0.1 | 0.1 | 0.065 | | 20.1 | 0.1 | 0.1 | 0.109 | | 20.1 | 0.1 | 0.1 | 0.141 | |
| 0.32% | 20.2 | 0.1 | 0.1 | 0.152 | | 20.0 | 0.1 | 0.0 | 0.449 | | 20.1 | 0.2 | 0.1 | 0.165 | |
| 1% | 20.1 | 0.4 | 0.0 | 0.465 | | 20.1 | 0.1 | 0.1 | 0.302 | | 20.1 | 0.1 | 0.1 | 0.081 | |
| 3.2% | 20.6 | 0.1 | 0.5 | <0.001 | | 20.3 | 0.2 | 0.3 | 0.011 | | 20.3 | 0.2 | 0.3 | 0.006 | |
| 10% | 27.5 | 5.5 | 7.4 | 0.002 | * | 23.2 | 3.8 | 3.2 | 0.019 | * | 25.5 | 8.2 | 5.5 | 0.039 | * |

**Table S2. Summary of lysis buffer dilutions for qPCR.** The average and standard deviation were calculated from 9 replicates. The $\Delta C_q$ is calculated by subtracting the average value for a given buffer concentration from the water condition (0%). A positive value indicates a cycle delay when adding the buffer. *P*-values were calculated by a 1-tailed unequal variance t-test compared to the water condition (0%). A * indicates a delay of at least 0.5 cycles and *P*-value <0.05. Non-detects were assigned a value of 40 cycles. PB = Qiagen PB Buffer.

| | Shield | | | | | Lysis | | | | | PB | | | | |
|---|---|---|---|---|---|---|---|---|---|---|---|---|---|---|---|
| | Avg | Std | ΔCq | p | * | Avg | Std | ΔCq | p | * | Avg | Std | ΔCq | p | * |
| 0% | 20.0 | 0.3 | | | | 20.0 | 0.3 | | | | 20.0 | 0.3 | | | |
| 0.03% | 20.0 | 0.1 | 0.0 | 0.445 | | 20.1 | 0.3 | 0.1 | 0.184 | | 20.1 | 0.2 | 0.1 | 0.101 | |
| 0.1% | 20.1 | 0.2 | 0.1 | 0.126 | | 20.0 | 0.1 | 0.0 | 0.340 | | 20.2 | 0.1 | 0.2 | 0.023 | |
| 0.32% | 20.2 | 0.2 | 0.2 | 0.047 | | 20.5 | 0.7 | 0.5 | 0.029 | * | 20.3 | 0.1 | 0.3 | 0.004 | |
| 1% | 32.4 | 8.5 | 12.4 | 0.001 | * | 22.3 | 0.7 | 2.3 | <0.001 | * | 21.2 | 0.1 | 1.2 | 0.000 | * |
| 3.2% | 40.0 | 0.0 | 20.0 | <0.001 | * | 40.0 | 0.0 | 20.0 | <0.001 | * | 40.0 | 0.0 | 20.0 | 0.000 | * |
| 10% | 40.0 | 0.0 | 20.0 | <0.001 | * | 40.0 | 0.0 | 20.0 | <0.001 | * | 40.0 | 0.0 | 20.0 | 0.000 | * |

**Table S3. Summary of ethanol-based buffer dilutions for LAMP.** The average and standard deviation were calculated from 9 replicates. The ΔTTP is calculated by subtracting the average value for a given buffer concentration from the water condition (0%). A positive value indicates a cycle delay. *P*-values were calculated by a 1-tailed unequal variance t-test compared to the water condition (0%). A * indicates a delay of at least 0.5 min and *P*-value <0.05. Non-detects were assigned a value of 46.7 min. VWB = Zymo Viral Wash Buffer; PE = Qiagen PE Buffer.

| | Ethanol | | | | | VWB | | | | | PE | | | | |
|---|---|---|---|---|---|---|---|---|---|---|---|---|---|---|---|
| | Avg | Std | ΔTTP | p | * | Avg | Std | ΔTTP | p | * | Avg | Std | ΔTTP | p | * |
| 0% | 6.3 | 0.2 | | | | 7.1 | 0.6 | | | | 7.1 | 0.6 | | | |
| 0.03% | 6.4 | 0.2 | 0.1 | 0.277 | | 7.2 | 0.7 | 0.1 | 0.420 | | 7.4 | 0.8 | 0.3 | 0.185 | |
| 0.1% | 6.5 | 0.4 | 0.1 | 0.182 | | 7.2 | 0.6 | 0.1 | 0.368 | | 7.2 | 0.8 | 0.1 | 0.380 | |
| 0.32% | 6.6 | 0.2 | 0.2 | 0.022 | | 7.1 | 0.7 | 0.0 | 0.494 | | 7.3 | 0.8 | 0.2 | 0.230 | |
| 1% | 6.9 | 0.6 | 0.5 | 0.019 | * | 7.2 | 0.7 | 0.1 | 0.333 | | 7.8 | 0.8 | 0.7 | 0.021 | * |
| 3.2% | 9.7 | 1.0 | 3.3 | <0.001 | * | 8.6 | 1.2 | 1.5 | 0.003 | * | 13.1 | 12.6 | 6.0 | 0.096 | |
| 10% | 46.7 | 0.0 | 40.3 | <0.001 | * | 46.7 | 0.0 | 39.6 | <0.001 | * | 43.5 | 9.6 | 36.4 | <0.001 | * |

**Table S4. Summary of lysis buffer dilutions for LAMP.** The average and standard deviation were calculated from 9 replicates. The ΔTTP is calculated by subtracting the average value for a given buffer concentration from the water condition (0%). A positive value indicates a cycle delay. *P*-values were calculated by a 1-tailed unequal variance t-test compared to the water condition (0%). A * indicates a delay of at least 0.5 min and *P*-value <0.05. Non-detects were assigned a value of 46.7 min. PB = Qiagen PB Buffer.

| | Shield | | | | | Lysis | | | | | PB | | | | |
|---|---|---|---|---|---|---|---|---|---|---|---|---|---|---|---|
| | Avg | Std | ΔTTP | p | * | Avg | Std | ΔTTP | p | * | Avg | Std | ΔTTP | p | * |
| 0% | 7.1 | 0.6 | | | | 7.1 | 0.6 | | | | 7.1 | 0.6 | | | |
| 0.03% | 6.7 | 0.5 | -0.4 | 0.078 | | 7.2 | 0.5 | 0.1 | 0.401 | | 7.0 | 0.8 | -0.1 | 0.338 | |
| 0.1% | 6.8 | 0.4 | -0.3 | 0.095 | | 7.2 | 0.4 | 0.1 | 0.331 | | 6.7 | 0.5 | -0.4 | 0.083 | |
| 0.32% | 38.2 | 16.9 | 31.1 | <0.001 | * | 8.4 | 1.0 | 1.3 | 0.002 | * | 6.3 | 0.5 | -0.8 | 0.005 | |
| 1% | 46.7 | 0.0 | 39.6 | <0.001 | * | 40.1 | 13.2 | 33.0 | <0.001 | * | 6.5 | 0.9 | -0.6 | 0.053 | |
| 3.2% | 46.7 | 0.0 | 39.6 | <0.001 | * | 46.7 | 0.0 | 39.6 | <0.001 | * | 44.9 | 5.4 | 37.8 | <0.001 | * |
| 10% | 46.7 | 0.0 | 39.6 | <0.001 | * | 46.7 | 0.0 | 39.6 | <0.001 | * | 46.7 | 0.0 | 39.6 | <0.001 | * |

### *Inhibitory effects on NA amplification curves*

We observed that qPCR reactions with lysis buffer (Fig. S7a, dashed lines) had lower amplification efficiency with each cycle compared with reactions lacking lysis buffer (Fig. S7a, solid lines). This experiment demonstrates that the presence of lysis buffer causes a delay in the $C_q$ and a reduction in the endpoint fluorescence intensity. Meanwhile, LAMP reactions with lysis buffer experienced an initiation delay, but the amplification rate and endpoint fluorescence intensity were not strongly affected (Fig. S7b).

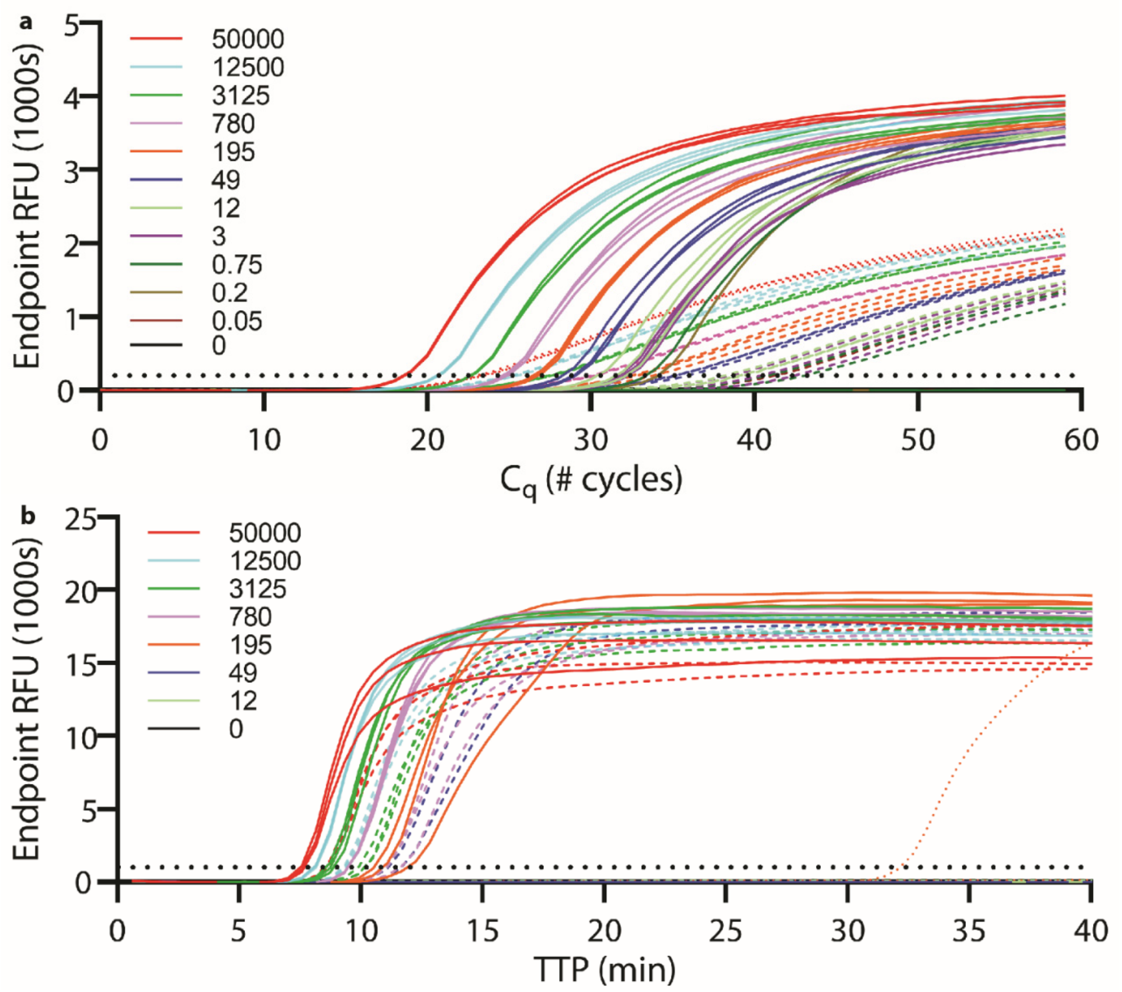


**Figure S7. (a) qPCR and (b) LAMP amplification curves with (dashed lines) or without (solid lines) Zymo Viral DNA/RNA Buffer for 4-fold dilutions of *E. coli* 23S rRNA gene copies. For** qPCR we used 1% lysis buffer and for LAMP we used 0.32% lysis buffer. Time-to-positive (TTP) threshold of 200 RFU for qPCR or 1000 RFU for LAMP is drawn as a dotted black line. Legend indicates the number of *E. coli* 23S rRNA gene copies/rxn. The qPCR amplification curves correspond to the experiment in Fig. 3 of the main text.

*TPW screen with qPCR and LAMP*

**Table S5. TPW screen with qPCR.** $\Delta C_q$ calculated by subtracting the “No additive” control from each condition.

| | Avg | Std | ΔCq | N |
|---|---|---|---|---|
| No additive | 20.09 | 0.01 | | 3 |
| water | 20.03 | 0.02 | -0.06 | 3 |
| Ethanol | 25.30 | 2.03 | 5.21 | 3 |
| Isopropanol | 24.54 | 2.66 | 4.44 | 3 |
| 1-butanol | N.D. | | | 0 |
| isopentanol | N.D. | | | 0 |
| 1-hexanol | N.D. | | | 0 |
| 1-heptanol | N.D. | | | 0 |
| 1-octanol | 23.63 | 1.10 | 3.54 | 2 |
| 1-nonanol | 20.07 | 0.07 | -0.03 | 3 |
| 1-decanol | 19.80 | 0.10 | -0.29 | 3 |
| 1-undecanol | 19.67 | 0.13 | -0.42 | 3 |
| 2-dodecanol | 19.81 | 0.03 | -0.28 | 3 |
| silicone oil | 19.86 | 0.19 | -0.23 | 3 |
| FC-40 | 20.15 | 0.17 | 0.06 | 3 |

**Table S6. TPW screen with LAMP.** ΔTTP calculated by subtracting the “No additive” control from each condition.

| | Avg | Std | ΔTTP | N |
|---|---|---|---|---|
| No additive | 6.54 | 0.05 | | 3 |
| water | 7.09 | 0.05 | 0.55 | 3 |
| Ethanol | N.D. | | | 0 |
| Isopropanol | N.D. | | | 0 |
| 1-butanol | N.D. | | | 0 |
| isopentanol | N.D. | | | 0 |
| 1-hexanol | N.D. | | | 0 |
| 1-heptanol | N.D. | | | 0 |
| 1-octanol | 11.18 | 2.44 | 4.63 | 3 |
| 1-nonanol | 7.41 | 0.06 | 0.87 | 3 |
| 1-decanol | 7.06 | 0.03 | 0.51 | 3 |
| 1-undecanol | 6.70 | 0.03 | 0.16 | 3 |
| 2-dodecanol | 6.43 | 0.05 | -0.11 | 3 |
| silicone oil | 6.49 | 0.02 | -0.06 | 3 |
| FC-40 | 6.64 | 0.04 | 0.09 | 3 |

*Solubility table and ethanol phase separation for TPW candidates*

**Table S7. Solubility table for two-phase wash (TPW) candidates**

| Candidate TPW | Solubility of TPW Candidate in water | Solubility of water in TPW Candidate |
|---|---|---|
| FC-40 [1] | <0.0050 % | < 0.0007 g / 100 g |
| Silicone oil [2] | Practically insoluble | 0.01 - 0.02 g / 100 g |
| 2-dodecanol [3] | Unknown | Unknown |
| 1-dodecanol [3] | 0.0004 g / 100 g | 3.0 g / 100 g |
| 1-undecanol [4] | 0.0015 g / 100 mL | 3.4 g / 100 g |
| 1-decanol [3] | 0.0037 g / 100 g | 3.6 g / 100 g |
| 1-nonanol [3] | 0.014 g / 100 g | 4.0 g / 100 g |
| 1-octanol [3] | 0.054 g / 100 g | 4.6 g / 100 g |
| 1-heptanol [3] | 0.174 g / 100 g | 5.4 g / 100 g |
| 1-hexanol [3] | 0.6 g / 100 g | 7.0 g / 100 g |
| Isopentanol [3] | 2.7 g / 100 g | 9.8 g / 100 g |
| 1-butanol [3] | 7.4 g / 100 g | 20.3 g / 100 g |
| Isopropanol | miscible | miscible |
| Ethanol | miscible | miscible |

**Table S8. Compounds were mixed at a 1:1 volume ratio.** A “2” denotes phase separation into 2 distinct phases whereas a “1” forms a single phase. VWB stands for Zymo Viral Wash Buffer, which contained 80% ethanol (v/v).

| | $H_2O$ | Ethanol | VWB |
|---|---|---|---|
| FC-40 | 2 | 2 | 2 |
| Silicone oil | 2 | 2 | 2 |
| 2-dodecanol | 2 | 1 | 1 |
| 1-undecanol | 2 | 1 | 1 |
| 1-octanol | 2 | 1 | 1 |

*Evaluating a 3-step centrifugation extraction with TPW*

We wanted to see whether in some cases the TPW could be considered as an alternative to the ethanol wash for removing lysis buffer. Exchanging the ethanol wash for a TPW could be useful for applications in which the starting sample is already relatively pure. For this experiment, we used the Zymo ZR kit, which only has three centrifugation steps: lysis (sample, shield, lysis buffer), wash (ethanol-based viral wash buffer), and elution (water). We either followed the manufacturer protocol or replaced the viral wash buffer with a dry spin, ethanol, or TPW (Fig. S8). We added 5 µL of the resulting eluent to 5 µL of LAMP reaction mix and amplified at 68C. Eluent from the manufacturer protocol amplified in 5.7 min. The dry spin did not amplify, which is expected because lysis buffer was not removed by any wash steps and lysis buffer is very inhibitory for LAMP. A 100% ethanol wash performed slightly better (earlier TTP) than the viral wash buffer and both 1-octanol and 2-dodecanol outperformed the wash buffer. Meanwhile, eluent from the silicone oil and FC-40 wash conditions did not amplify. A dPCR experiment on heavy dilutions of the eluent show similar recovery for all conditions, with a slight reduction for the silicone oil wash. This demonstrates that 1-octanol and 2-dodecanol remove lysis buffer from the column. The simplicity of a 3-step protocol (bind, wash, elute) is compatible with point-of-care devices (few steps), and could be useful for applications with relatively clean samples.

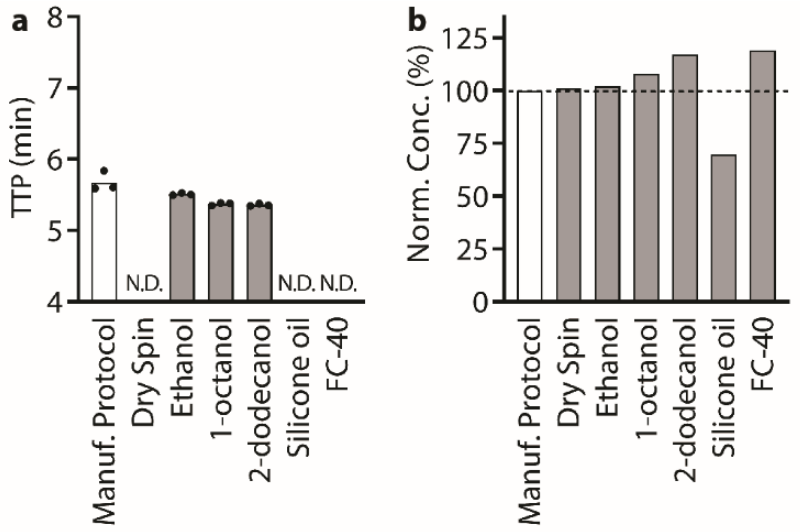


**Figure S8. Evaluation of TPW as a potential alternative to ethanol-based viral wash buffer in a Zymo ZR kit.** (a) LAMP reaction with 2x dilution of eluent and (b) dPCR reaction with 100x dilution of eluent. Bars represent the average of technical LAMP triplicates or merged duplicate dPCR measurements. We ran 7 extractions (1 silica column x 7 conditions) and same eluent was used LAMP and dPCR reactions. No template controls (n=3) and samples marked N.D. were not detected within 40 min.

*Evaluating a low-carryover, high-yield MagBead protocol*

The manufacturer protocol for the Zymo Quick-DNA/RNA Viral MagBead Kit led to significant extraction buffer carryover (as shown in Figs. 7- 8). To improve NA yield with the added TPW, we performed the initial TPW aspiration, waited at least 1 min, and aspirated any remaining TPW. This second aspiration collected a few microliters of residual buffer that dripped down from the walls of the tube or from the magnetic beads. To reduce carryover of all buffers, we also applied this 1 min wait and secondary aspiration to all steps (lysis/binding buffer, wash buffers). We evaluated this modified protocol for different TPWs and the results are shown in Fig. S9. At high dilutions of eluent, there were no visible indicators of inhibition for any of the samples. Our modified protocol greatly reduced carryover overall, such that qPCR began to work even at low dilutions (whereas when run using the standard manufacturer protocol we saw inhibition). The addition of the TPW further improved LAMP at low dilutions. Finally, NA recovery improved to 75-100%, achieving our original goal.

When inhibitors are a major concern and time is not an issue, we recommend performing the MagBead protocol with secondary aspirations on each step, adding a 10-min dry step, and adding the TPW. For an approach balancing performance and assay time, we recommend following the manufacturer protocol, replacing the 10-min dry step with the TPW, and adding a secondary aspiration step just prior to the elution.

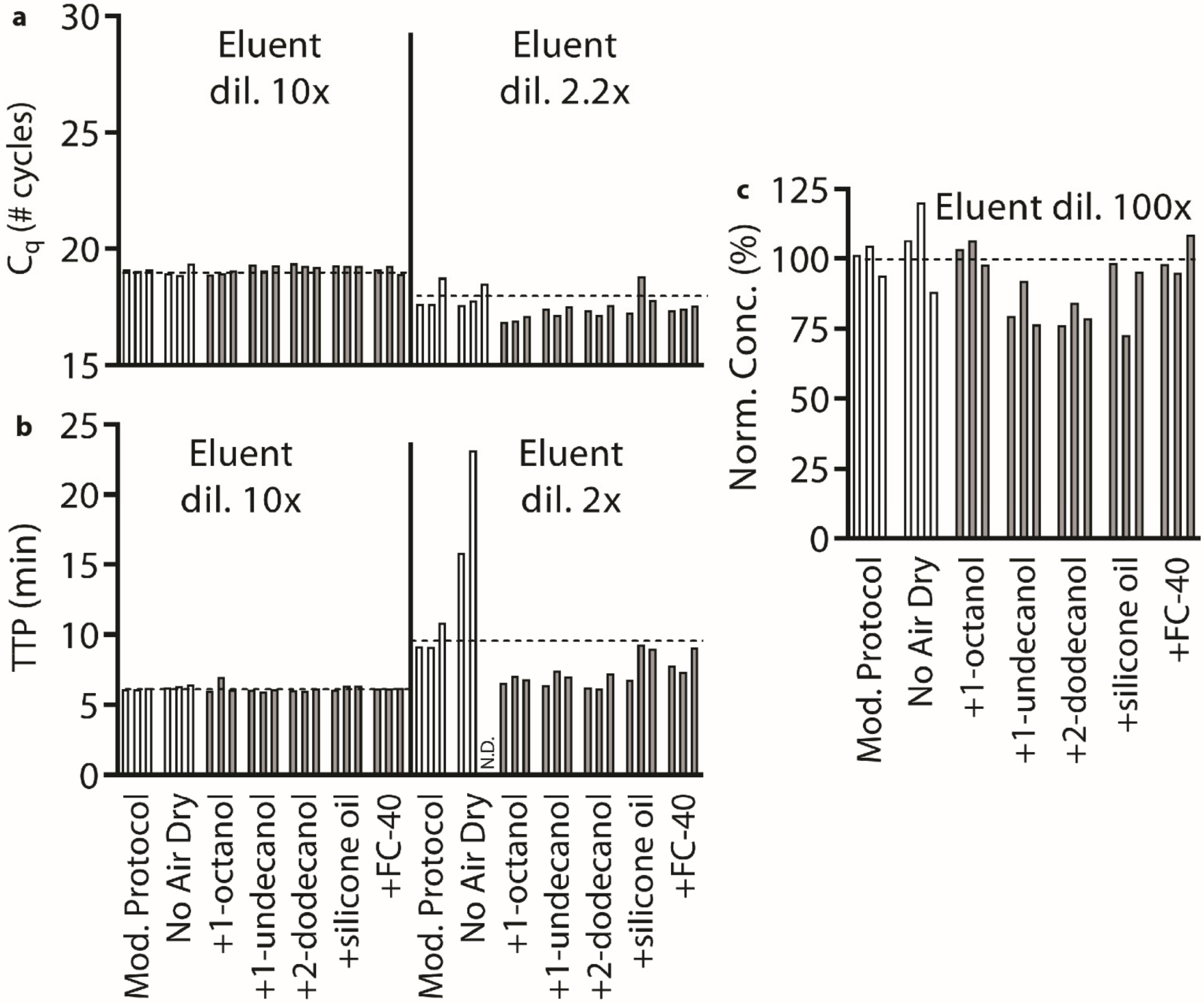


**Figure S9. Evaluation of a modified Zymo Quick-DNA/RNA Viral MagBead Kit for reduced carryover with and without TPW by (a) qPCR, (b) LAMP, or (c) dPCR.** All conditions were performed with a modified protocol for high NA yield when combined with TPW. MagBead extractions were performed on 2.5 x $10^6$ λ phage DNA copies. Low and high eluent dilutions evaluated by qPCR and LAMP. A 100x eluent dilution into dPCR shows high yield with TPW. Bars represent single qPCR and LAMP reactions or merged duplicate dPCR measurements. We ran 21 extractions (3 magnetic-bead extractions x 7 conditions) and the same eluent was used in qPCR, LAMP, and dPCR analyses.

*Statistical analysis methods*

Confidence intervals were calculated assuming the populations to be normally distributed and using a t statistic. For buffer inhibition experiments, statistical analysis was performed by a 1-tailed unequal variance t-test (N=9) comparing the water condition (control) to each buffer concentration ($H_1$: the mean is delayed). For the subsequent experiments, we used a 2-tailed unequal variance t-test ($H_1$: the means are different). Non-detects were assigned the maximum possible $C_q$ measurement of 40 cycles or a TTP of 46.7 min to indicate the lack of amplification. Although this approach introduces some bias into the analysis, we believe this is the best representation for handling non-detects (other alternatives include excluding the non-detects or assigning non-detect values to the average of those that amplified).

There are many potential sources of experimental variation (e.g. column-to-column, day-to-day generation of master mix, buffer dilutions, and pipetting errors), and we tried to control for these by running triplicates for different variables (buffer/MM dilutions, columns, technical qPCR/LAMP assays). *A priori*, we would have assumed our independent variables to be differences in buffer dilutions or differences among columns, and we expected that our technical replicates would display a narrow distribution. Instead, we observed large variations among technical replicates (e.g. 2 out of 3 amplify). Because large variations appear at the level of the technical replicate, we treated each technical replicate as an independent sample in our statistical analysis.

Familywise error rate across the reported statistical analyses was not controlled (e.g. Bonferroni correction). All data have been made publicly available and, to strengthen the findings of this study, we encourage further replication and validation, as there are numerous different potential applications and variables to examine (e.g. sample matrices, extraction kits, sequencing, etc.).

*References*


1. 3M Fluorinert™ FC-40 Electronic Liquid. *MatWeb Material Property Data* http://www.matweb.com/search/DataSheet.aspx?MatGUID=2072a809f9ca4d529b1d136660736f81
2. Silicone Fluids: Stable, Inert Media. *Gelest, Inc* (2004).
3. Barton, Allan FM. Alcohols with Water in *Solubility Data Series Volume 15* (2013).
4. 1-Undecanol. *GESTIS Substance Database* http://www.dguv.de/ifa/gestis-database
